# Transcriptional Profiles of Long Non-coding RNA and mRNA in Sheep Mammary Gland During Lactation Period

**DOI:** 10.3389/fgene.2020.00946

**Published:** 2020-09-25

**Authors:** Weihao Chen, Xiaoyang Lv, Yue Wang, Xinjun Zhang, Shanhe Wang, Zahid Hussain, Ling Chen, Rui Su, Wei Sun

**Affiliations:** ^1^College of Animal Science and Technology, Yangzhou University, Yangzhou, China; ^2^Animal Hospital of Yangzhou University, Yangzhou University, Yangzhou, China; ^3^Animal Science and Veterinary Medicine Bureau of Suzhou City, Suzhou, China; ^4^Suzhou Taihu Dongshang Sheep Industry Development Co., Ltd., Suzhou, China; ^5^Joint International Research Laboratory of Agriculture and Agri-Product Safety of Ministry of Education of China, Yangzhou University, Yangzhou, China; ^6^College of Veterinary Medicine, Yangzhou University, Yangzhou, China

**Keywords:** sheep, lactation, lncRNA, mRNA, RNA-Seq

## Abstract

Sheep milk and related products have been growing in popularity around the world in recent years. However, the sheep milk industry is limited by low milk yield, and the molecular regulators of ovine lactation remain largely unknown. To investigate the transcriptomic basis of sheep lactation, RNA-Sequencing was used to explore the expression profiles of lncRNA and mRNA of the mammary gland in Hu sheep at three key time points during the lactation stage: 5 days before the expected date of parturition perinatal period (PP), 6 days after parturition early lactation (EL), and 25 days after parturition peak lactation (PL). A total of 1111, 688, and 54 differentially expressed (DE) lncRNAs as well as 1360, 660, and 17 DE mRNAs were detected in the EL vs PP, PL vs PP, and PL vs EL comparisons, respectively. Several prominent mRNAs (e.g., *CSN1S1*, *CSN1S2*, *PAEP*, *CSN2*, *CSN3*, and *COL3A1*) and lncRNAs (e.g., LNC_018483, LNC_005678, LNC_012936, and LNC_004856) were identified. Functional enrichment analysis revealed that several DE mRNAs and target genes of DE lncRNAs were involved in lactation-related pathways, such as MAPK, PPAR, and ECM–receptor interaction. This study enhances our understanding of how transcriptomic profiles change during the lactation period and pave the way for future studies examining sheep lactation.

## Introduction

Given that one of the major challenges of the milk industry is dietary structure and nutritional diversity, there is a constant need for milk producers to improve milk products to meet the diverse demands of consumers. These demands have included milk with higher nutritional content in addition to sheep milk and related products, as sheep milk is richer in fat, protein, lactose, vitamins, and trace elements compared with cow and goat milk (Suarez-Vega et al., 2017; Balthazar et al., 2019). However, the sheep milk industry is limited by the low milk yield of sheep. The production of milk is a complex trait regulated by several biological processes and shows moderate heritability (Marti et al., 2013; Banos et al., 2019); therefore, the use of genomic selection to improve the selection of milk traits has become increasingly important for the sheep milk industry.

With the rapid growth in RNA-Sequencing (RNA-Seq) technologies, transcriptomic approaches have become widely used to understand complex traits, such as reproductive traits (Zhang et al., 2019), milk traits (Billa et al., 2019), and muscle growth (Xiang et al., 2018), and have increased our understanding of non-coding RNAs (ncRNAs). Among ncRNAs, long non-coding RNAs (lncRNAs), which were previously considered junk RNAs, have been found to be involved in individual neurodevelopment (Zhang et al., 2020), cell cycle regulation (Luo et al., 2020), and cancer (Wang et al., 2018; Chen et al., 2019). For example, Yoon et al. (2012) found that lincRNA p21 can directly inhibit the translation of *Jun B* and *CTNNB1*. lncRNA SNHG6 can regulate cell proliferation by participating in the translation and splicing of microRNAs (miRNAs) in cancer (Meng et al., 2019).

In addition, lncRNAs can act as competitive endogenous RNAs (ceRNAs) to affect the expression of mRNAs (Xu et al., 2020). Growing evidence has revealed that lncRNAs can both directly and indirectly regulate mRNAs. Hence, the role of lncRNAs and mRNAs in various important biological processes has received increased attention by researchers. For example, Ma et al. (2018) profiled mRNA and lncRNA expression in sheep that differed in the deposition of fat in their tails and identified several DE mRNAs and lncRNAs, suggesting that they may play crucial roles in the deposition of tail fat. Ji et al. (2020) characterized the mRNA and lncRNA expression profiles in dairy goats from different lactation periods and found that the expression of lncRNAs was tied to mammary gland development and lactation. However, little comparative research of the lncRNAs and mRNAs in sheep lactation has been conducted. In addition, how lncRNAs and mRNAs interact during sheep lactation has not been characterized.

Hu sheep, a Chinese native sheep breed, have drawn much attention for their excellent lactation performance and high prolificacy (two births per year, 2–3 lambs per birth, Lv et al., 2019); therefore, Hu sheep provide an ideal animal model for dairy sheep breeding. However, the molecular mechanism underlying lactation in sheep is largely unknown, yet elucidating this molecular mechanism is important for increasing our knowledge of sheep lactation. Considering the essential role that mammary glands play in lactation (Azagra-Boronat et al., 2020; Hooper et al., 2020), we extracted mammary gland biopsies from Hu sheep during three key time points of lactation (PP, EL, and PL) and used RNA-Seq to study the expression profiles of lncRNAs and mRNAs. Our study improves our understanding of the potential roles of lncRNAs and mRNAs during lactation and promises to aid more in-depth studies on the breeding of dairy sheep.

## Materials and Methods

### Sample Collection

All experimental sheep were kept under similar conditions and fed at Zhenjiang Wan Shan Hong Bian Agricultural Co., Ltd. (Zhenjiang, Jiangsu Province, China) with detailed litter records. A total of 10 first-time pregnant ewes close to their pregnancy date were selected for sampling. Approximately 1.5-g mammary gland parenchyma biopsies were extracted from each ewe by surgical biopsy at three key points during the lactation period: 5 days before the expected date of parturition PP, 6 days after parturition (EL), and 25 days after parturition (PL) according to the lactation curve of Hu sheep (Li et al., 2015). Mammary gland biopsies were snap-frozen in liquid nitrogen and stored at −80°C. Mammary gland biopsies of three ewes with the same litter size were then selected for RNA extraction.

All of the aforementioned experimental procedures were approved by the Experimental Animal Welfare and Ethics Institute of Animal Science, Yangzhou University. Generally, all efforts were taken to minimize pain and discomfort to animals while conducting these experiments.

### RNA Extraction and Transcriptome Sequencing

RNA was extracted from the mammary gland biopsies with TRIzol reagent per the manufacturer’s instructions. The quality of isolated RNA was examined using an RNA Nano 6000 Assay Kit, and RNA integrity Number (RIN) was checked by Agilent 2100 bioanalyzer as the threshold of RIN ≥ 8.0.

An Epicenter Ribo-Zero^TM^ Kit was used to remove rRNA, and the lncRNA library was constructed using the NEBNext^®^ Ultra^TM^ RNA Library Prep Kit for Illumina^®^ per the manufacturer’s instructions, followed by dilution of the lncRNA library to a concentration of 1 ng/μL. For mRNA sequencing, total RNA was firstly poly-A-selected followed by fragmentation of RNA into small pieces. The cleaved RNA fragments were reverse transcribed to cDNA and ligated with Illumina adapters using the NEBNext^®^ Ultra^TM^ RNA Library Prep Kit for Illumina^®^ per the manufacturer’s instructions. The libraries were then fractionated on agarose gel; short fragments (approximately 200 bp in length) were excised and amplified by PCR. Finally, the RNA libraries were sequenced on PE150 strategy (pair end 150 bp) using Illumina HiSeq^TM^ 2500 by Beijing Novogene Technology Co., Ltd.

### Processing of Reads

Raw reads were obtained in the FASTQ format. They were arranged according to the number of reads, base amount, and Q30/20 (the proportion of read bases whose error rate is less than 0.1/1%). Among the raw reads that were generated by sequencing, low-quality reads that included adapters, reads that contained N (wherein the proportion of bases that could not be identified >10%), and low-quality reads (base with sQ ≤ 5 accounts for more than 50% of the entire reads, those with quality scores < Q20) were removed. Clean reads were then obtained and mapped to the *Ovis aries* reference genome (Oar_v4.0) using HISAT2 (Kim et al., 2015); StringTie (Pertea et al., 2015) was used to assemble the transcripts of mRNAs.

Coding and non-coding RNA candidates from the transcripts were distinguished with Coding-Non-Coding-Index (CNCI, Sun et al., 2013), coded potential calculator-2 (CPC2, Kong et al., 2007), and Pfam-scan (PFAM, El-Gebali et al., 2019) software after transcriptome assembly. Non-coding RNA candidates with lengths >200 nt, and exon numbers ≥2 were identified as candidate lncRNAs.

### Analyses of Differential Expression

The expected number of Fragments Per Kilobase of transcript sequence per Million fragments sequenced (FPKM, Trapnell et al., 2010) was used to estimate the expression levels of candidate lncRNA and mRNA transcripts; transcripts were used for the subsequent analysis as the threshold of FPKM ≥ 0.5. Multiple comparisons were conducted to identify differentially expressed (DE) lncRNAs and DE mRNAs among the PP, EL, and PL groups using DEseq (Wang et al., 2010). lncRNAs and mRNAs were considered significantly DE as the threshold of | log2Foldchange| >1 and *q*-value (*p*-values adjusted by Benjamini and Hochberg’s approach) <0.05.

### Target Gene Prediction and DE Interaction Network Analysis

To better understand lncRNA function, we identified predicted cis- and trans-target genes. Coding genes located 100 kb upstream and downstream of the corresponding lncRNAs were considered cis-target genes. In addition, we calculated Pearson correlation coefficients between the expression level of coding genes and corresponding lncRNAs. Coding genes were considered trans-target genes if the | correlation| ≥ 0.95.

Based on the target gene prediction, the interactions between DE lncRNAs and DE mRNAs were used to construct the DE interaction network using Cytoscape v3.7.2 software (Shannon et al., 2003).

### Gene Ontology (GO) and Kyoto Encyclopedia of Genes and Genomes (KEGG) Enrichment

Genes were functionally annotated using the DE mRNAs and target genes of the DE lncRNAs based on the results of the DE analysis and the predicted target genes. GOseq R library (Young et al., 2010) and KOBAS (KO-Based Annotation System, Xie et al., 2011) programs were used for GO enrichment and KEGG pathway enrichment analyses, respectively. The number of genes enriched in GO and KEGG was determined, followed by a Fisher’s exact test with a false discovery rate (FDR) multiple test correction to assess statistical significance (*P* < 0.05).

### Validation of Sequencing Data

Six DE mRNAs and six DE lncRNAs were randomly selected. The housekeeping gene *RPL-19* was selected as the reference gene, the sequences of the selected lncRNAs are shown in Supplementary Table S1, and the primers were designed using Primer Premier 5 software (Supplementary Table S1).

Total RNA was extracted from the mammary gland biopsies from three ewes processed for RNA-Seq using a total RNA extraction kit for animal tissue. TRIzol was used to dissolve the mammary gland biopsies. The quantity and quality of total RNA were monitored using 1.5% agarose gel electrophoresis (*U* = 150 V;10 min) and ultraviolet spectrophotometry, respectively. The A260/280 ratios (1.8–2.0) of the RNA samples were all 1.9 to 2.0, which showed that the extracted total RNA was of acceptable purity with no contamination or degradation. Therefore, the RNA preparations were deemed fit for use in the follow-up experiments, and so were stored at -80°C until use.

The first strand of cDNA was prepared using a PrimeScript^TM^ RT Reagent Kit per the manufacturer’s instructions. The PCR thermocycler program was as follows: 37°C for 15 min, followed by 85°C for 5 s. The reaction mixture contained 1.0 μL PrimeScript RT Enzyme, 1.0 μL random 6-mers, 4.0 μL 5 × PrimeScript Buffer (for Real Time), 1.0 μL total RNA, and 13 μL RNase-free ddH_2_O (total volume, 20 μL). Prior to storage at -80°C, the standard working concentration of cDNA was 200 ng/μL. The quality of cDNA was evaluated by housekeeping gene amplification, and cDNA were stored at -20°C until use.

Real-time quantitative polymerase chain reaction (RT-qPCR) was performed with cDNA in triplicate to validate the reliability of sequencing data following the SYBR Green I method. RT-qPCR conditions were as follows: 1 cycle of 95°C for 15 min, followed by 40 cycles of 95°C for 10 s and 60°C for 30 s. The dissociation curve was analyzed after amplification. The melting temperature (Tm) peak observed at 65–95°C on the dissociation curve was used to determine the specificity of the PCR amplification.

The 2^–Δ^ ^Δ^ ^*Ct*^ method (Livak and Schmittgen, 2001) was used to calculate the relative expression level. The results were shown as the fold change of relative expression level (mean ± standard error of the mean) using GraphPad Prism 6 software.

## Results

### Overview of the Sequencing Data

The average numbers of raw reads of each group were 119,203,825 (PP), 103,187,784 (EL), and 103,117,901 (PL); the average numbers of clean reads of each group were 116,442,803 (PP), 100,365,485 (EL), and 99,916,856 (PL); and the average mapping rates of PP, EL, and PL were 81.71, 83.23, and 80.03%, respectively. Details are shown in Table 1.

**TABLE 1:** Summary of the sequencing data.

Sample name	Raw reads	Clean reads	Clean bases	Error rate (%)	Q20 (%)	Q30 (%)	GC content (%)
EL1	85,892,976	83,397,464	12.51G	0.02	98.21	94.87	54.23
EL2	122,553,262	119,604,884	17.94G	0.02	97.62	93.42	54.86
EL3	101,117,114	98,094,108	14.71G	0.02	97.75	93.45	52.16
PL1	81,546,644	79,336,108	11.9G	0.02	98.09	94.62	55.07
PL2	93,928,750	91,352,292	13.7G	0.02	97.8	93.7	54.9
PL3	133,878,310	129,062,170	19.36G	0.02	95.43	89.94	53.57
PP1	123,985,202	121,100,554	18.17G	0.02	97.63	93.63	53.62
PP2	127,128,826	124,208,970	18.63G	0.02	98.41	95.14	54.89
PP3	106,497,448	104,018,886	15.6G	0.02	97.44	93.14	52.96

*EL, PL, and PP represent early lactation, peak lactation, and perinatal period, respectively. Error rate% represents average error rate of sequencing of the single base.*

**FIGURE 1:**
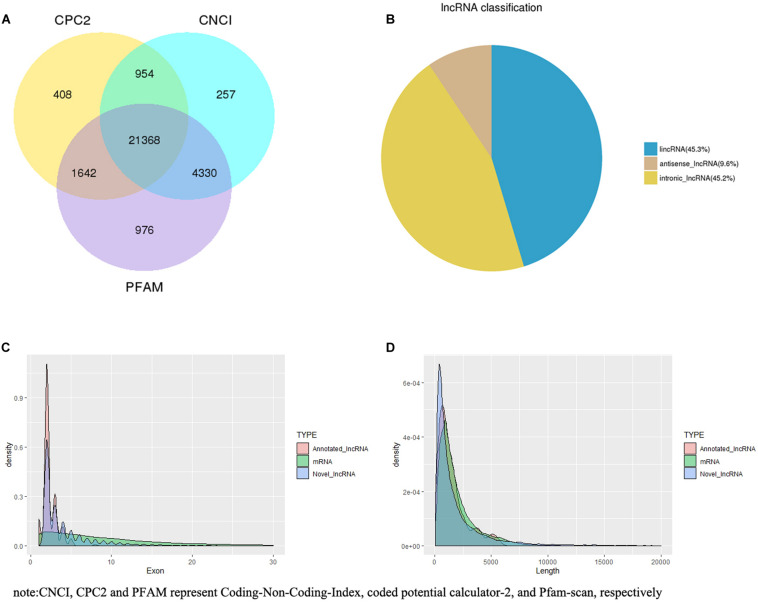
lncRNA filter/classification and exon/length distribution of identified lncRNAs and mRNAs. **(A)** Filter of identified lncRNAs. **(B)** Classification of identified lncRNAs. **(C)** Exon number distribution of identified lncRNAs and mRNAs. **(D)** Length distribution of identified lncRNAs and mRNAs.

Based on CPC2, CNCI, and PFAM, 21,368 novel lncRNAs were identified (Figure 1A), within which 45.27, 45.18, and 9.55% were identified as lincRNA, intronic lncRNA, and antisense lncRNA, respectively (Figure 1B). Additionally, 240 annotated lncRNAs and 22,823 mRNAs were detected. Overall, 21,608 lncRNAs (21,368 novel lncRNAs and 240 annotated lncRNAs) and 22,823 mRNAs were screened for in-depth analyses.

Most of the lncRNAs had two or three exons (2.32 on average), which was markedly fewer than the number of exons in mRNAs (10.06 on average) (Figure 1C). The length of the lncRNAs was primarily between 200 and 1,000 nt, and the average length was 2,164.41 nt. The length of mRNAs was primarily distributed within the range of 500–3,000 bp, and the average length was 1,992.07 bp (Figure 1D).

### Expression Profiles of lncRNAs and mRNAs

**FIGURE 2:**
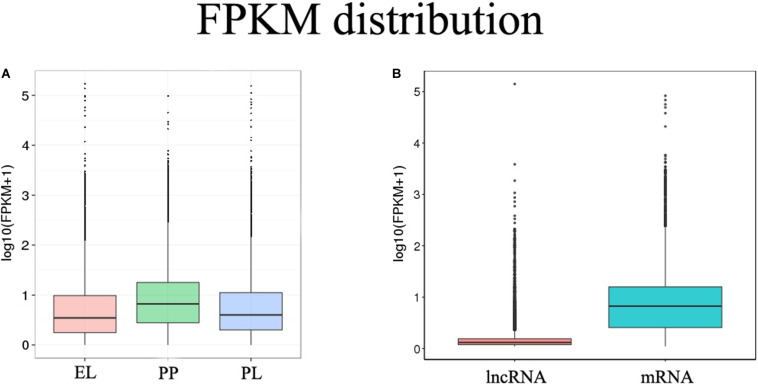
FPKM distribution. **(A)** FPKM distribution of different groups. **(B)** FPKM distribution of identified lncRNAs and mRNAs.

FPKM was performed to estimate the expression levels of lncRNAs and mRNAs. mRNA had a significantly higher expression level than lncRNA (*q* < 0.01), and the expression of mRNA was higher during the PP than during the EL and PL (Figure 2).

**FIGURE 3:**
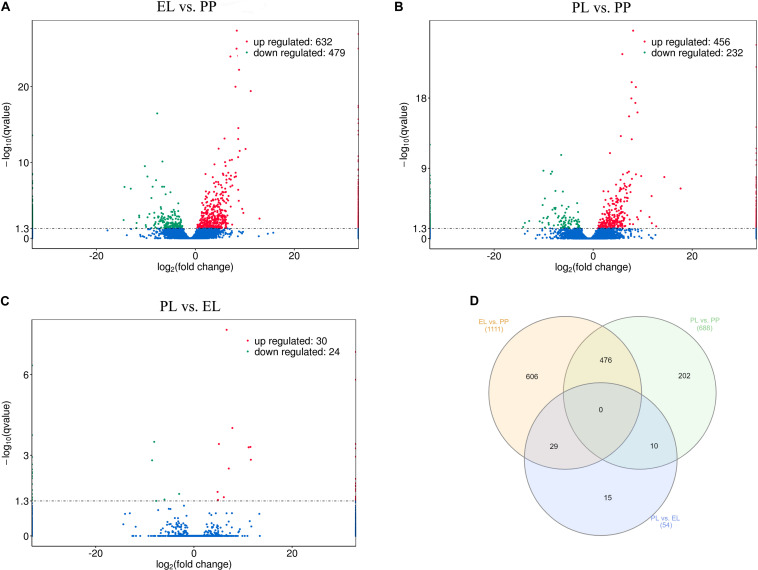
Differentially expressed (DE) lncRNAs. **(A)** Volcano plot of differentially expressed lncRNAs in early lactation (EL) vs perinatal period (PP), where red and green represent upregulation or downregulation, respectively, same below. **(B)** Volcano plot of DE lncRNAs in peak lactation (PL) vs PP. **(C)** Volcano plot of DE lncRNAs in PL vs EL. **(D)** Venn diagram of DE lncRNAs, where orange, green, and blue represent EL vs PP, PL vs PP, and PL vs EL, respectively.

DEseq was conducted based on FPKM to study DE lncRNAs and DE mRNAs. A total of 1111, 688, and 54 DE lncRNAs were detected in EL vs PP (Figure 3A), PL vs PP (Figure 3B), and PL vs EL (Figure 3C), respectively. The Venn diagrams of the DE lncRNAs between different comparison groups are shown in Figure 3D. Details are provided in Supplementary Table S2.

**FIGURE 4:**
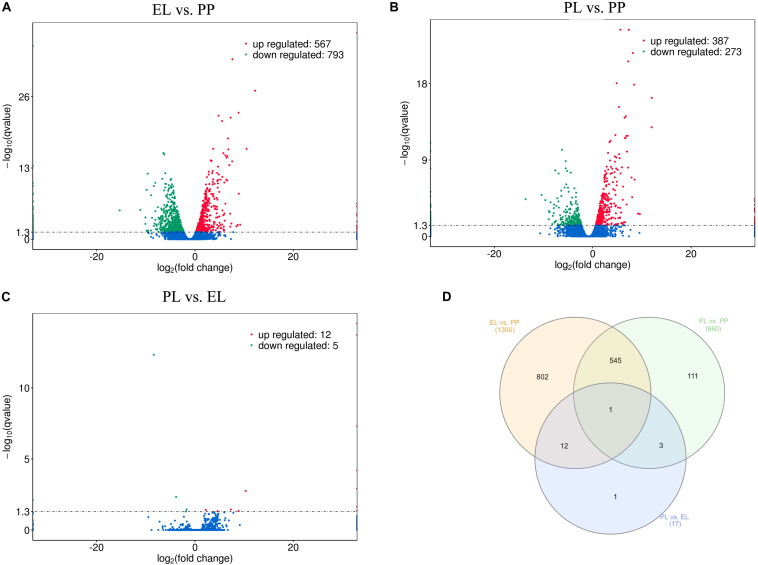
Differentially expressed (DE) mRNAs. **(A)** Volcano plot of DE mRNAs in early lactation (EL) vs perinatal period (PP). **(B)** Volcano plot of DE mRNAs in peak lactation (PL) vs PP. **(C)** Volcano plot of DE mRNAs in PL vs EL. **(D)** Venn diagram of DE mRNAs.

A total of 1360, 660, and 17 DE mRNAs were detected in EL vs PP (Figure 4A), PL vs PP (Figure 4B), and PL vs EL (Figure 4C), respectively. The Venn diagrams of the DE mRNAs between different comparison groups are shown in Figure 4D. *COL3A1* (ENSOART00000017961) was DE in all three comparisons. Details are provided in Supplementary Table S2.

**FIGURE 5:**
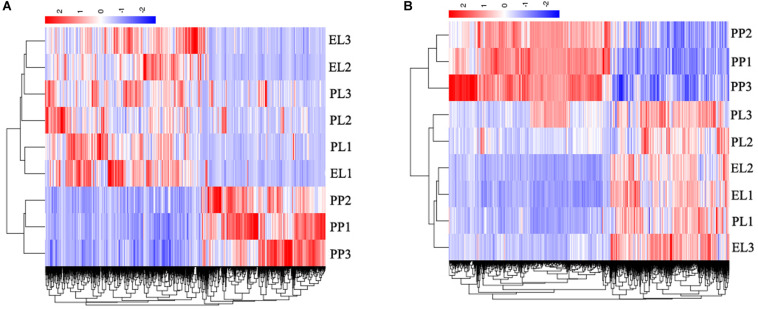
Heatmap of differentially expressed (DE) lncRNAs **(A)** and DE mRNAs **(B)**.

The heat maps of DE lncRNAs (Figure 5A) and DE mRNAs (Figure 5B) revealed different expression patterns between PP and lactation period (EL and PL); however, the difference between EL and PL was not significant.

### Target Gene Prediction and DE Interaction Network Analysis

Overall, 19, 153 cis-target genes of 19,129 corresponding lncRNAs and 15, 462 trans-target genes of 5,443 corresponding lncRNAs were predicted. Based on the DE analysis and the mRNA transcripts of genes, a total of 546 DE lncRNAs were found to cis-regulate 430 DE mRNAs, and 290 DE lncRNAs were found to trans-regulate 1,105 DE mRNAs (Supplementary Table S3). The DE interaction network was built on DE lncRNA–DE mRNA interactions. Furthermore, the number of connections of each candidate was quantified. Among DE lncRNAs, the top three most connected (lncRNA with the highest number of connections between corresponding mRNA) were LNC_005678 (212), LNC_012936 (201), and LNC_004856 (197); among DE mRNAs, the top three most connected (mRNA with the highest number of connections between corresponding lncRNA) were ENSOART00000000850 (*CRABP1*, 23), ENSOART00000018937 (22), and ENSOART00000017132 (*DCN*, 21). The high-resolution DE interaction network is shown in Supplementary Figure S1.

### GO and KEGG Enrichment Analyses

GO and KEGG enrichment analyses were conducted using the DE mRNAs and target genes of the DE lncRNAs.

**FIGURE 6:**
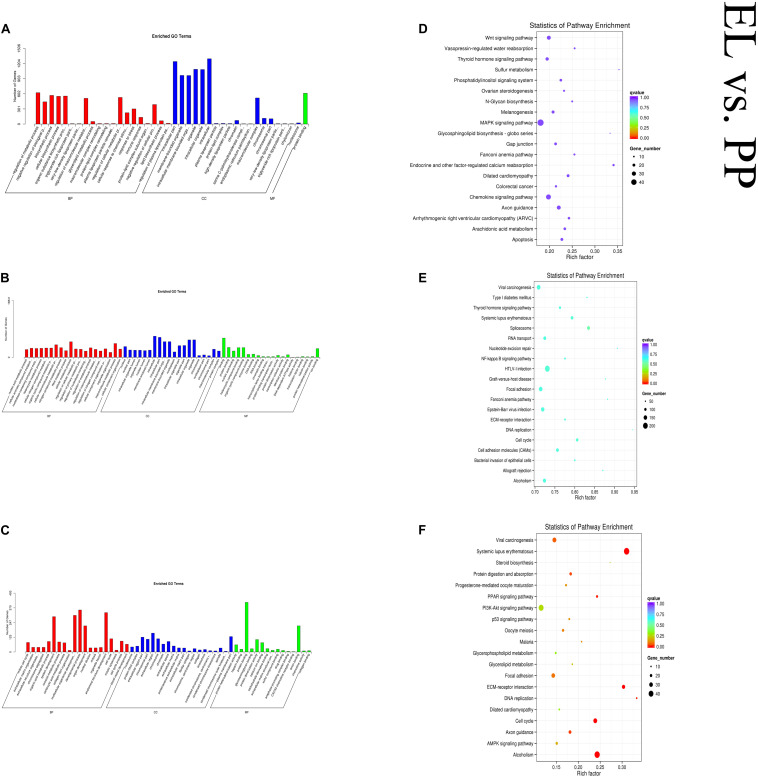
Top annotated GO terms of cis-target genes **(A)** and trans-target genes **(B)** of differentially expressed (DE) lncRNAs and DE mRNAs **(C)** and top enriched KEGG pathways of cis-target genes **(D)** and trans-target genes **(E)** of DE lncRNAs and DE mRNAs **(F)** in the early lactation (EL) vs perinatal period (PP).

In the EL vs PP comparison, DE mRNAs were significantly enriched in 166 GO terms. The top enriched GO terms were mitotic cell cycle (GO:0000278), nucleosome (GO:0000786), and protein heterodimerization activity (GO:0046982) in biological process (BP), cellular component (CC), and molecular function (MF), respectively. The cis-target genes of the DE lncRNAs were significantly enriched in 56 GO terms. The top enriched GO terms were regulation of metabolic process (GO:0019222), intracellular part (GO:0044424), and protein binding (GO:0005515) in BP, CC, and MF, respectively. The trans-target genes of the DE lncRNAs were significantly enriched in 762 GO terms. The top enriched GO terms were nucleic acid metabolic process (GO:0090304), nucleus (GO:0005634), and binding (GO:0005488) in BP, CC, and MF, respectively. DE mRNAs were significantly enriched in 31 KEGG pathways. The cis-target genes of the DE lncRNAs were significantly enriched in 15 KEGG pathways. The trans-target genes of the DE lncRNAs were significantly enriched in 22 KEGG pathways, within which pathways related to lactation were enriched, such as cell cycle (oas04110) and PPAR (oas03320). Figure 6 shows some of the top enriched GO terms and KEGG pathways, and the details are provided in Supplementary Table S4.

**FIGURE 7:**
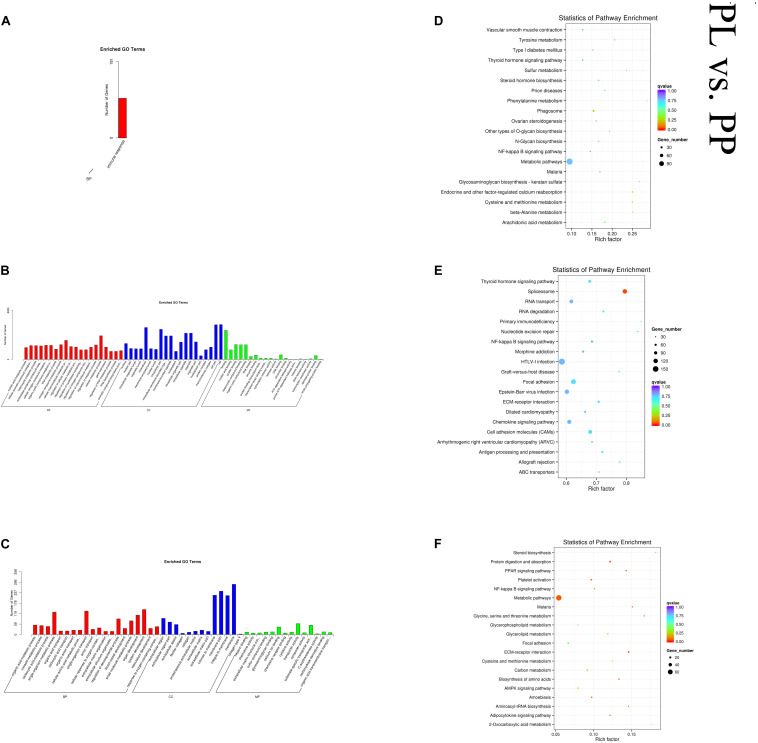
Top annotated GO terms of cis-target genes **(A)** and trans-target genes **(B)** of differentially expressed (DE) lncRNAs and DE mRNAs **(C)** and top enriched KEGG pathways of cis-target genes **(D)**, and trans-target genes **(E)** of DE lncRNAs and DE mRNAs **(F)** in peak lactation (PL) vs perinatal period (PP).

In the PL vs PP comparison, DE mRNAs were significantly enriched in 64 GO terms. The top enriched GO terms were organic acid metabolic process (GO:0006082), extracellular region (GO:0005576), and heparin binding (GO:0008201) in BP, CC, and MF, respectively. The cis-target genes of the DE lncRNAs were significantly enriched in only one GO term: immune response (GO:0006955). The trans-target genes of the DE lncRNAs were significantly enriched in 576 GO terms. The top enriched GO terms were nucleic acid metabolic process (GO:0090304), nucleus (GO:0005634), and binding (GO:0005488) in BP, CC, and MF, respectively. DE mRNAs were significantly enriched in 23 KEGG pathways. The cis-target genes of the DE lncRNAs were significantly enriched in 15 KEGG pathways, and the trans-target genes of the DE lncRNAs were significantly enriched in 11 KEGG pathways, within which pathways related to lactation were enriched, such as cell adhesion molecules (oas04514), metabolic (oas01100), and several metabolism-related pathways. Figure 7 shows some of the top enriched GO terms and KEGG pathways, and the details are provided in Supplementary Table S4.

**FIGURE 8:**
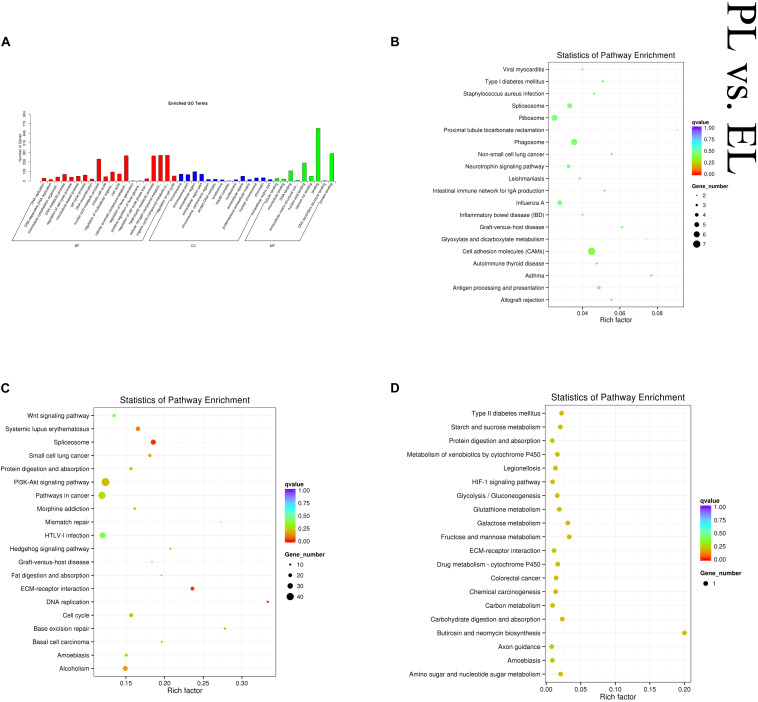
Top annotated GO terms of trans-target genes of differentially expressed (DE) lncRNAs **(A)** and top enriched KEGG pathways of cis-target genes **(B)** and trans-target genes **(C)** of DE lncRNAs and DE mRNAs **(D)** in peak lactation (PL) vs early lactation (EL).

In the PL vs EL comparison, DE mRNAs and cis-target genes of the DE lncRNAs were found to be significantly enriched in no GO terms, and trans-target genes of the DE lncRNAs were significantly enriched in 59 GO terms. The top enriched GO terms were DNA replication (GO:0006260), chromosome (GO:0005694), and tubulin binding (GO:0015631) in BP, CC, and MF, respectively. DE mRNAs were significantly enriched in three KEGG pathways. The cis-target genes of the DE lncRNAs were significantly enriched in 14 KEGG pathways, and the trans-target genes of the DE lncRNAs were significantly enriched in 24 KEGG pathways, within which pathways related to lactation were enriched, such as cell cycle (oas04110) and ECM–receptor interaction (oas04512). Figure 8 shows some of the top enriched GO terms and KEGG pathways, and the details are provided in Supplementary Table S4.

### Validation of Sequencing Data

**FIGURE 9:**
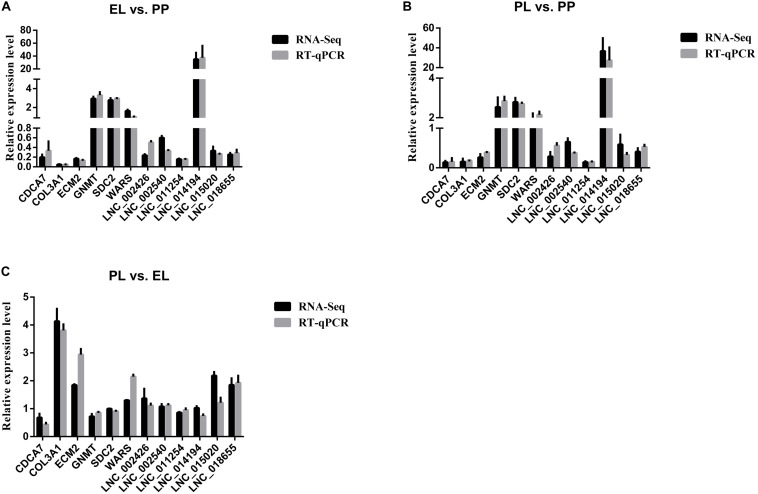
Comparisons of the results of the RNA-seq and RT-qPCR analyses of DE lncRNAs and mRNAs in early lactation (EL) vs perinatal period (PP) **(A)**, peak lactation (PL) vs PP **(B)**, and PL vs EL **(C)**.

The melting curve chart and melting peak chart of RT-qPCR are shown in Supplementary Table S2, which indicated the accuracy and repeatability of our RT-qPCR data. The comparison of the expression level of DE lncRNAs and DE mRNAs selected for verification of the accuracy of sequencing between RNA-Seq and RT-qPCR are shown in Figure 9. The results indicated that selected lncRNAs and mRNAs showed similar expression patterns between RNA-Seq and RT-qPCR, suggesting the reliability of our sequencing data.

## Discussion

The mammary gland is a key organ related to lactation in mammals, and the milk yield is largely controlled by the mammary epithelial cells (MECs). During pregnancy and the lactation period, MECs go through three stages: proliferation, differentiation, and apoptosis (Tsugami et al., 2020; Zhou et al., 2020). From the beginning of pregnancy to the end of the PP, the mammary gland is further developed and rapid proliferation of the MECs takes place. After parturition, the MECs differentiate into secretory cells, regulate lactation, and remain stable during lactation. During PL to late lactation, the milk yield begins to decrease, and the apoptosis of MECs begins (Rios et al., 2016). Hence, we selected three key time points in the development of MECs to study the molecular mechanisms underlying sheep lactation: PP (proliferation), EL (differentiation), and PL (beginning of apoptosis). Numerous studies have found an association between birth size, parity, and milk yield in cows (Kamal et al., 2014; Pinedo et al., 2020), goats (Zumbach et al., 2008), and sheep (David et al., 2008; Dettori et al., 2018): specifically, the milk yield tends to increase with birth size and parity. To enhance the reliability of the sequencing data, mammary gland biopsies from first-time pregnant ewes with the same litter size were selected for the final sequencing.

### Transcriptomic Profiles

The average mapping rate in our study was 81.66%, and 21,608 lncRNAs and 22,823 mRNAs were identified. 1111, 688 DE lncRNAs and 1360, 660 DE mRNAs were detected in the EL vs PP and PL vs PP comparisons, respectively, indicating a clear difference in the expression profiles of lncRNAs and mRNAs between the perinatal and lactation periods. In PL vs EL, 54 DE lncRNAs and 17 DE mRNAs were identified. The numbers of DE lncRNAs and mRNAs in this comparison were far fewer than those observed in other comparison groups (EL vs PP and PL vs PP). Our results are consistent with previous studies in dairy goats (Guan et al., 2020) and cows (Lin et al., 2019), implying that the transcriptomic state in the mammary gland during lactation may be a widespread characteristic of livestock.

*COL3A1* was found to be DE in all three comparisons. Specifically, the expression level of *COL3A1* rapidly decreased from PP to the lactation period and then remained steady. Previous studies of cancer have demonstrated the role that *COL3A1* can play in the proliferation of cancer cells (Qiu et al., 2015; Xu et al., 2018), Furthermore, the overexpression of *COL3A1* can lead to a significant decrease in the phosphorylation of the AKT/mTOR pathway (Kuivaniemi and Tromp, 2019), a pathway that is important for lactation (Wang et al., 2014). This evidence suggests that *COL3A1* may play an important role in the sheep MEC cycle and would make a prime candidate for future research.

Among the DE mRNAs, the top five with the highest expression levels were *CSN1S1*, *CSN1S2*, *PAEP*, *CSN2*, and *CSN3* (according to the average FPKM values) and showed similar expression profiles (increasing sharply then decreasing slightly during the three stages). Four tightly linked casein genes, *CSN1S1*, *CSN1S2*, *CSN2*, and *CSN3* (Bovenhuis and Weller, 1994), were particularly noteworthy. Among livestock, sheep are the least studied with respect to the effect of these four casein genes on lactation. No association was revealed between polymorphisms at the *CSN3* loci and milk yield in East Friesian sheep (Staiger et al., 2010). However, several studies have revealed that these genes play key roles in determining milk-related traits, such as protein rate, fat rate, and milk yield in dairy cows (Rodney et al., 2016) and goats (Cardona et al., 2016). One potential explanation for these inconsistencies is that the animal models differ in their properties. Nevertheless, given their critical roles in lactation, it is likely that *CSN1S1*, *CSN1S2*, *CSN2*, and *CSN3* play a role in sheep lactation; however, additional work is needed to confirm this possibility. One of the DE lncRNAs, LNC_018483, whose cis-target genes were *CSN1S1*, *CSN1S2*, and *CSN2*, also showed a similar expression pattern to these genes. Consequently, future work should determine whether LNC_018483 mediates the effects of casein genes on sheep lactation. Consistent with previous studies in cattle (Yang et al., 2016) and sheep (Suarez-Vega et al., 2015), *PAEP*, which encodes the major milk whey β-lactoglobulin protein, showed a higher expression level in the lactation period than in the non-lactation period. *PAEP* is essential for lactation and could potentially explain the transcriptomic features of sheep milk protein.

Higher expression levels of *DGAT2*, *STAT1*, *STAT2*, and *STAT5A*, candidate genes that regulate milk fat and protein (Arun et al., 2015; Liu et al., 2020), were detected in the PP. Our results are inconsistent with previous studies in dairy cows (Bionaz and Loor, 2011; Karlengen et al., 2012; Bovenhuis et al., 2016) that revealed a positive correlation between the expression level of these genes and milk yield. Instead, our results demonstrated that *DGAT2*, *STAT1*, *STAT2*, and *STAT5A* may be negatively related to milk fat and lactose in sheep; however, additional research is needed to test this hypothesis. No significant changes in the expression of *DGAT1*, *STAT3*, *STAT6*, and *STAT5B* genes were detected. Little research has been conducted on these genes in sheep; thus, whether these genes play a role in sheep lactation remains unclear.

We also compared the expression level of several classic cell cycle-related transcriptional regulators during different periods, including members of the CDK, CCK, PPAR, Blc-2, and DR families. A rapid downregulation in expression was observed in most members of the CDK (*CDK1*, *CDKL1*, and *CDKN3*), CCN (*CCNA2*, *CCNB*2, *CCNE2*, *CCNF*, *CCNY*, and *CCNYL1*), and PPAR families (*PPARG*) in the lactation period compared with the non-lactation period. Several studies have demonstrated that these genes can inhibit the proliferation of cancer cells, muscle cells, and MECs (Small et al., 2014; Yeger and Perbal, 2016; Sarek et al., 2019). The proliferation of MECs during the lactation period and their expression profiles suggest that they may inhibit the proliferation of MECs in sheep. A sharp increase (from PP to EL) followed by a slight decrease (from EL to PL) during the three periods was detected in the expression level of apoptosis-related genes, such as *DR1* and several members of the SLC family (*SLC26A2*, *SLC35C2*). Given the critical role of these genes in the apoptosis of cells (Morioka et al., 2018; Qiu et al., 2018; Scalise et al., 2018), we hypothesize that the decline in lactation may begin during PL in sheep. Furthermore, members of the CDK and CCN families (Yeger and Perbal, 2016; Sarek et al., 2019) also showed non-significant up-regulation in PL vs EL, which also is consistent with our hypothesis.

Most of the top DE lncRNAs (according to fold change) of the three comparisons were predicted to be target members of the FAM family, such as LNC_012374 (1st in EL vs PP, cis-target FAM160B1), LNC_008239 (2nd in EL vs PP, trans-target FAM124A), and LNC_001741 (3rd in EL vs PP, trans-target FAM167B), LNC_020336 (2nd in PL vs PP, trans-target FAM184A), LNC_001741 (1st in EL vs PL, trans-target FAM167B), and LNC_008239 (2nd in EL vs PL, trans-target FAM124A). FAM, novel regulators in the caspase family, have been overwhelmingly confirmed to play a critical role in the intracellular regulation of cell apoptosis in cancer (Mannherz et al., 2006). Despite the tremendous interest in FAM in cancer cells, little research has been conducted to assess the influence of FAM on the MEC cycle. Our new data and findings contribute to our understanding of the molecular mechanisms underlying the sheep MEC cycle.

### Functional Enrichment of DE mRNAs and lncRNAs

In all three comparisons, GO annotation showed that the target genes of DE lncRNAs and DE mRNAs were primarily involved in the extracellular region, metabolism, and cell cycle. These results suggest that DE mRNAs and lncRNAs contribute, both directly and indirectly, to diverse lactation-related processes, highlighting the complex metabolic and extracellular changes that the mammary gland undergoes throughout sheep lactation.

The KEGG pathway enrichment analysis of target genes of the DE lncRNAs revealed that the cell cycle and MAPK signaling pathway were significantly enriched for both the EL vs PP and PL vs PP comparisons (lactation vs non-lactation). In dairy cows, the MAPK signaling pathway can downregulate the expression of IGF-β protein in bovine MECs; in addition, the expression of IGF-β protein is closely related to the apoptosis of bovine MECs (Fleming et al., 2007; Yu et al., 2012). We thus hypothesized that a regulatory relationship between lncRNA, the MAPK signaling pathway, and MECs may also exist in sheep.

The KEGG pathway enrichment analysis of DE mRNAs revealed that two crucial lactation-related pathways—the PPAR and ECM-receptor interaction signaling pathways—were significantly enriched. The PPAR signaling pathway has been reported to be associated with the vitality and proliferation of MECs in goats (Shi et al., 2013). Similarly, considerable evidence has shown that the ECM can directly or indirectly contribute to cell differentiation, proliferation, and apoptosis (Zhang et al., 2016). Hence, from our perspective, PPAR and ECM–receptor interaction signaling pathways may also affect the cycle of MECs in sheep. However, several key pathways in lactation, such as mTOR and PI3K-AKT, were not significantly enriched in our study. Thus, our findings suggest that proteins related to lactation are synthesized by other pathways during the sheep lactation period.

### DE Interaction Network

To further understand the functions of lncRNAs and mRNAs in sheep lactation, we constructed a DE lncRNA–DE mRNA interaction network that contained 79 DE lncRNAs and 1,214 DE mRNAs. LNC_005678, LNC_012936, and LNC_004856 had the highest numbers of connections in the network and targeted 27, 29, and 16 members of the SLC family, respectively. All of these lncRNAs were predicted to target *COL3A1*, which was DE in all three comparisons. The top DE mRNA with the highest number of connections in the network was ENSOART00000017132 (*DCN*). A core component of proteoglycan, *DCN* can regulate many important cellular biological processes, including cell proliferation, differentiation, apoptosis, and carcinogenesis (Xu et al., 2016). There is a high probability that these lncRNAs and mRNAs interact closely and thus act as key regulators in MECs; thus, future work should focus on the potential roles that these RNAs play in sheep lactation.

## Conclusion

RNA-Seq data from mammary gland biopsies extracted during the perinatal, EL and PL periods were used to gain insight into the expression profiles of lncRNAs and mRNAs during lactation in sheep. Several potential candidate DE mRNAs (e.g., *CSN1S1*, *CSN1S2*, *PAEP*, *CSN2*, *COL3A1*, CDK, CCN, FAM, and SLC), lncRNAs (e.g., LNC_012374, LNC_008239, and LNC_001741), and mRNA–lncRNA pairs (e.g., *CSN1S1*, *CSN1S2*, *CSN2-*LNC_018483, *COL3A1*-LNC_005678, LNC_012936, and LNC_004856) were detected. Functional enrichment analysis revealed that several DE mRNAs and target genes of DE lncRNAs were involved in lactation-related pathways, such as MAPK, PPAR, and ECM–receptor interaction. Our findings can path the way for future studies of lncRNAs and mRNAs involved in lactation as well as help elucidate the molecular mechanisms underlying lactation in sheep.

## Data Availability Statement

The datasets presented in this study can be found in online repositories. The names of the repository/repositories and accession number(s) can be found below: https://www.ncbi.nlm.nih.gov/, PRJNA634610.

## Ethics Statement

The animal study was reviewed and approved by Experimental Animal Welfare and Ethical of Institute of Animal Science, Yangzhou University. Written informed consent was obtained from the owners for the participation of their animals in this study.

## Author Contributions

WS, WC, YW, XL, XZ, SW, and LC designed the research. WC, YW, XL, XZ, ZH, and RS collected the data. WC analyzed the data and drafted the manuscript. WS and SW revised the final manuscript. All authors read and approved the final manuscript.

## Conflict of Interest

RS was employed by company employed by company Suzhou Taihu Dongshang Sheep Industry Development Co., Ltd., China. The remaining authors declare that the research was conducted in the absence of any commercial or financial relationships that could be construed as a potential conflict of interest.

## Supplementary Material

FIGURE S1 High-resolution differentially expressed mRNA–differentially expressed lncRNA interaction network.

FIGURE S2 Melting curve chart and melting peak chart of RT-qPCR.

TABLE S1 Primer information of RT-qPCR and sequences of the selected lncRNAs for sequencing validation.

TABLE S2 Differentially expressed mRNAs and lncRNAs for three comparisons between different time points of the lactation stage.

TABLE S3 Details of the differentially expressed mRNAs–differentially expressed lncRNAs interaction network.

TABLE S4 Functional enrichment of differentially expressed mRNAs and target genes of differentially expressed lncRNAs for three comparisons between different time points of the lactation stage (early lactation vs perinatal period; peak lactation vs perinatal period; and peak lactation vs early lactation).
