# Supplementary material for: Transcriptional Profiles of Long Non-coding RNA and mRNA in Sheep Mammary Gland During Lactation Period
Source: Front Genet. 2020 Sep 25;11:946. doi: 10.3389/fgene.2020.00946 (PMC7546800; doi:10.3389/fgene.2020.00946)
Supplement: FIGURE S1 — High-resolution differentially expressed mRNA–differentially expressed lncRNA interaction network. [file Data_Sheet_1.PDF]

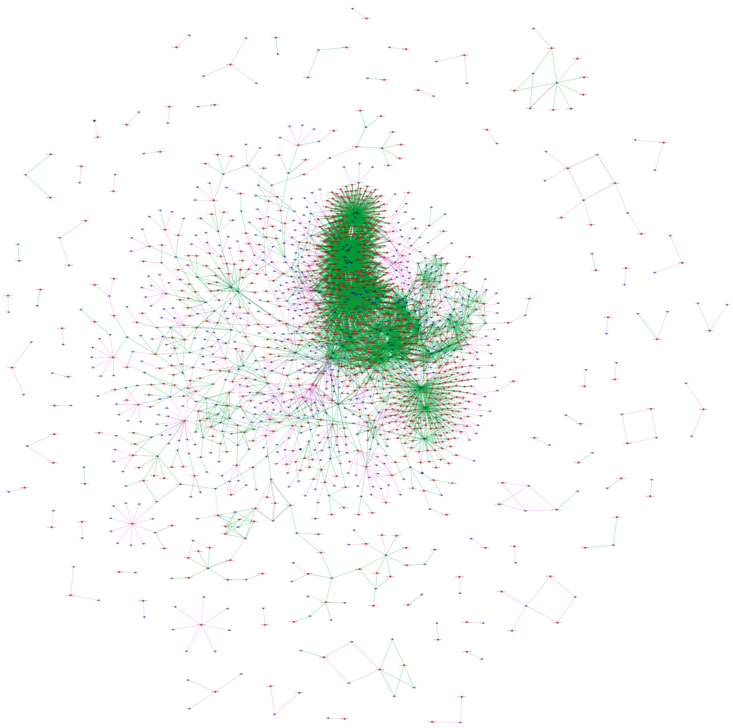

The differentially expressed interaction network, where the "V" shape (blue) and circle (red) represent lncRNAs and mRNAs, respectively; dashed lines (purple) and solid lines (green) represent cis-target and trans-target genes, respectively.
