# Supplementary material for: Transcriptional Profiles of Long Non-coding RNA and mRNA in Sheep Mammary Gland During Lactation Period
Source: Front Genet. 2020 Sep 25;11:946. doi: 10.3389/fgene.2020.00946 (PMC7546800; doi:10.3389/fgene.2020.00946)

Melting curve chart of lnc/mRNAs selected for RT-qPCR

LNC_002426

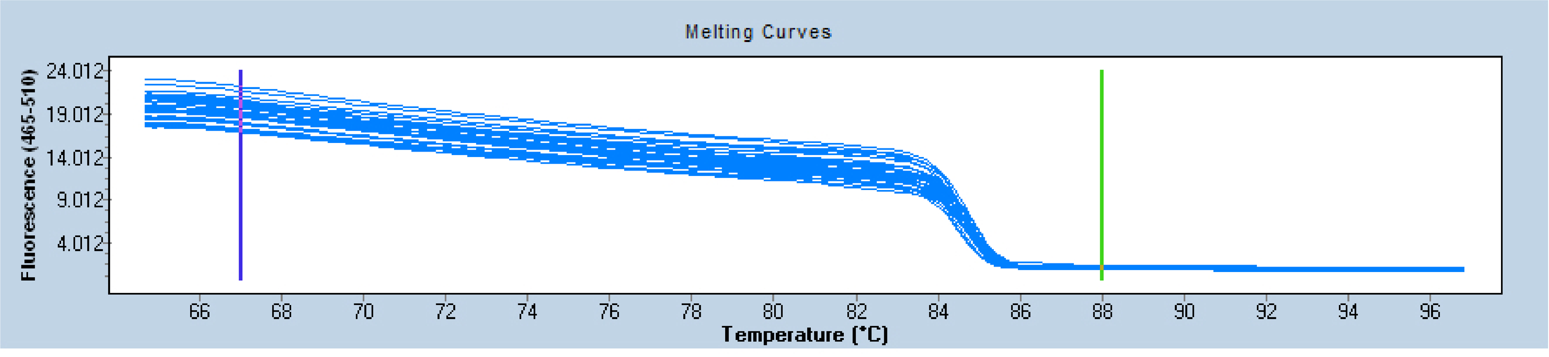

LNC_002540

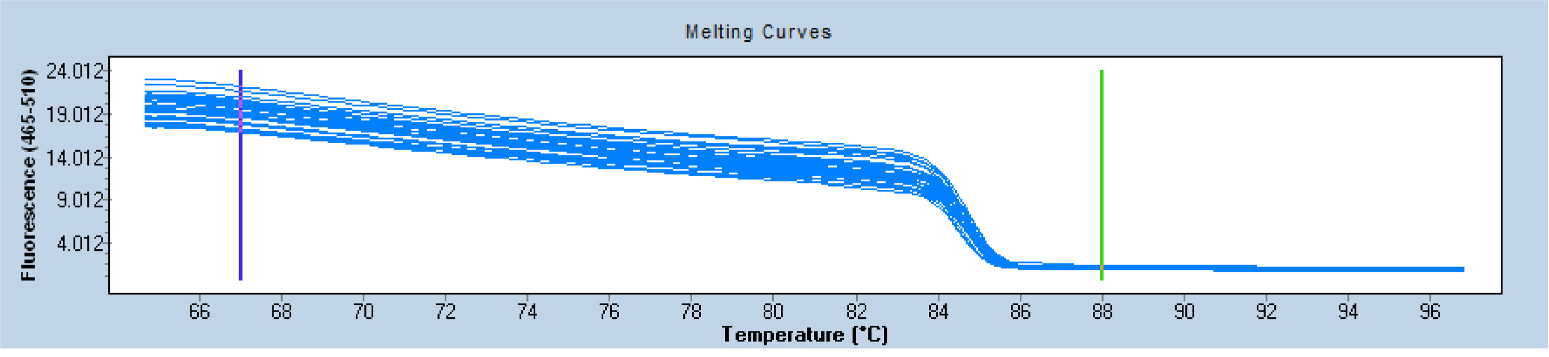

LNC_011254

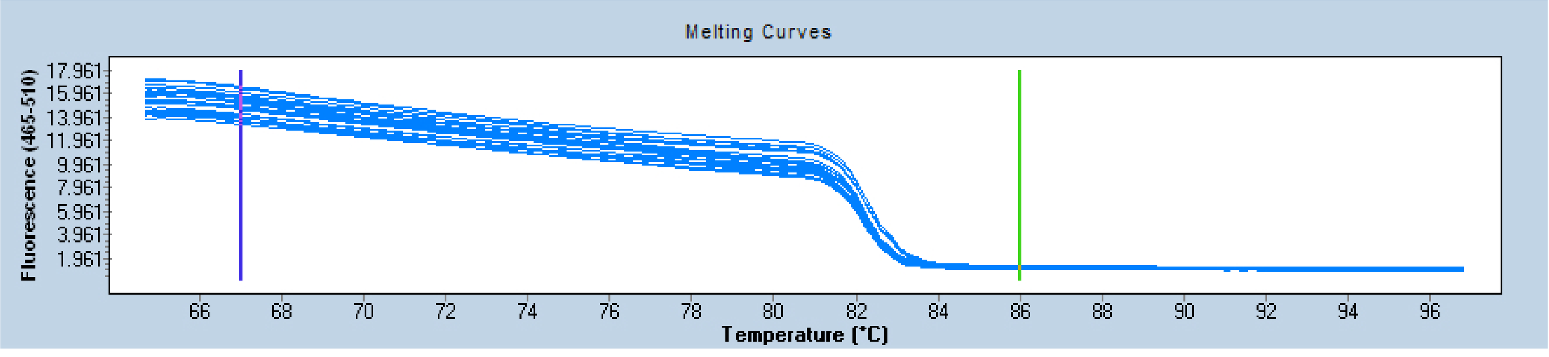

LNC_014194

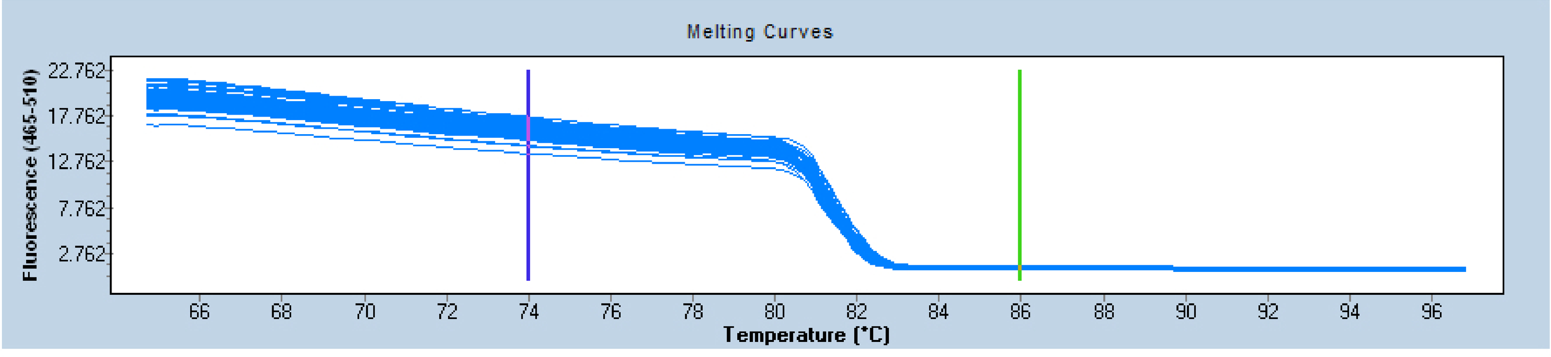

LNC_015020

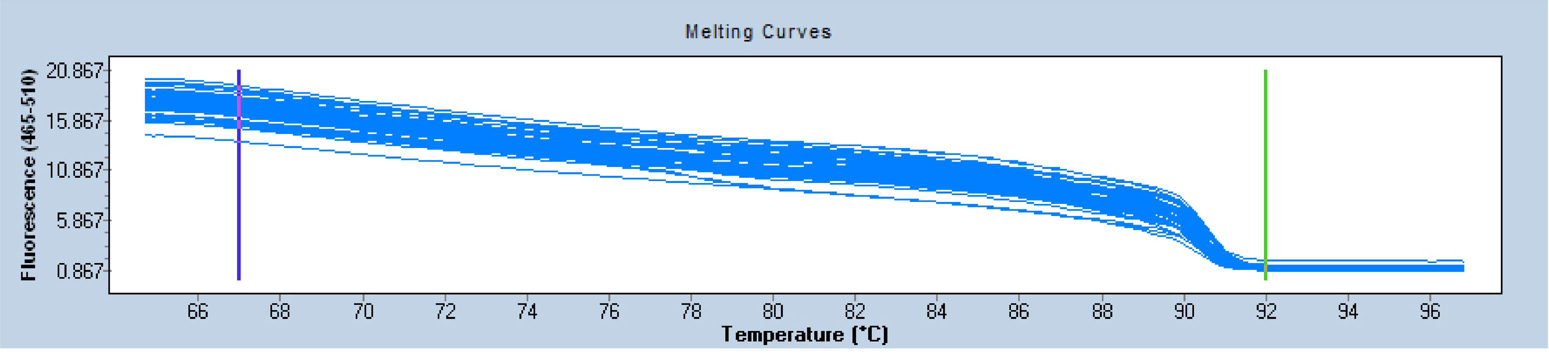

LNC_018655

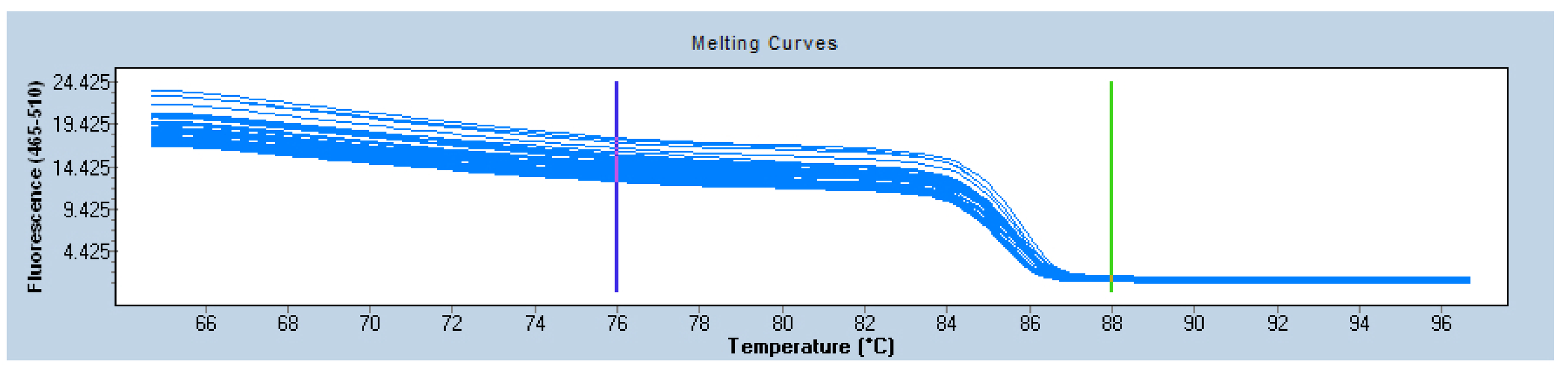

CDCA7

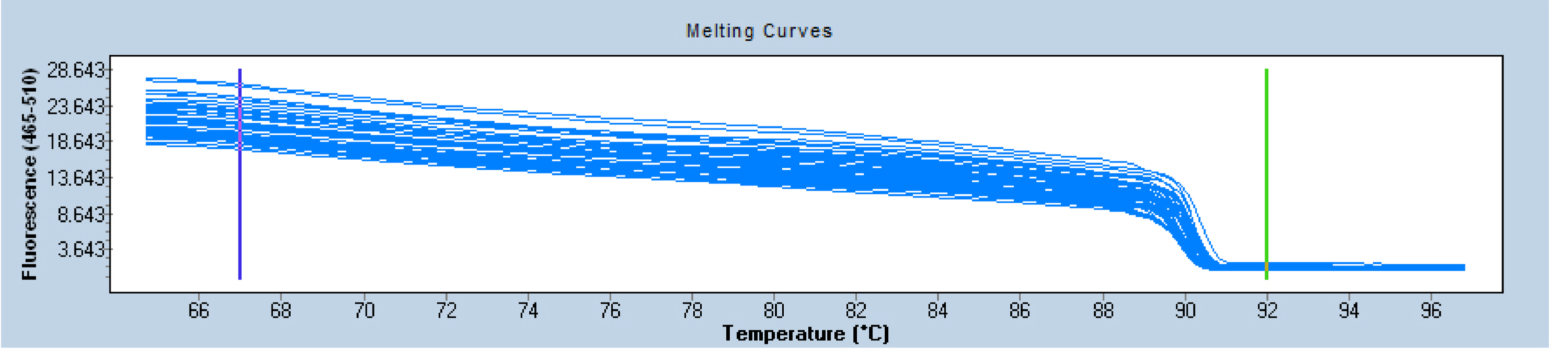

COL3A1

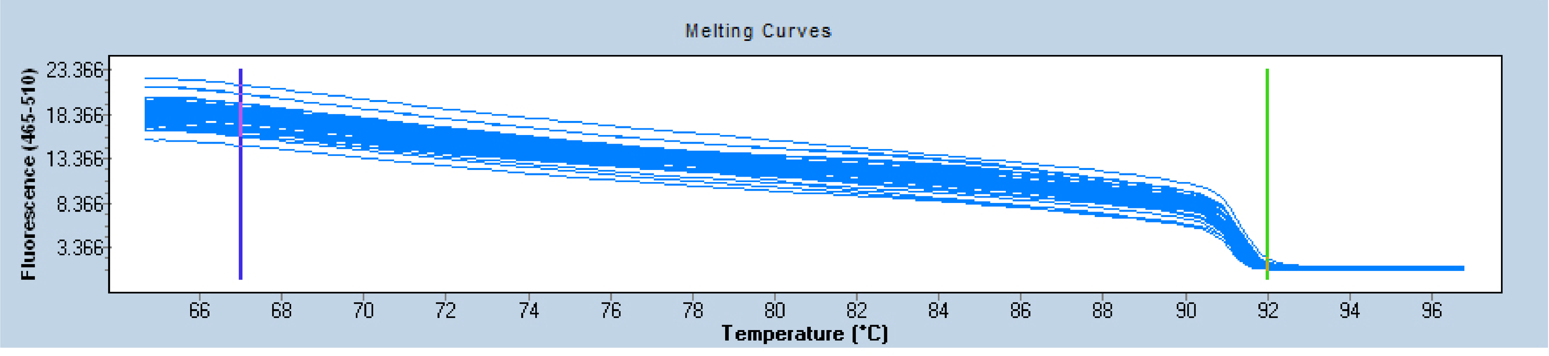

ECM2

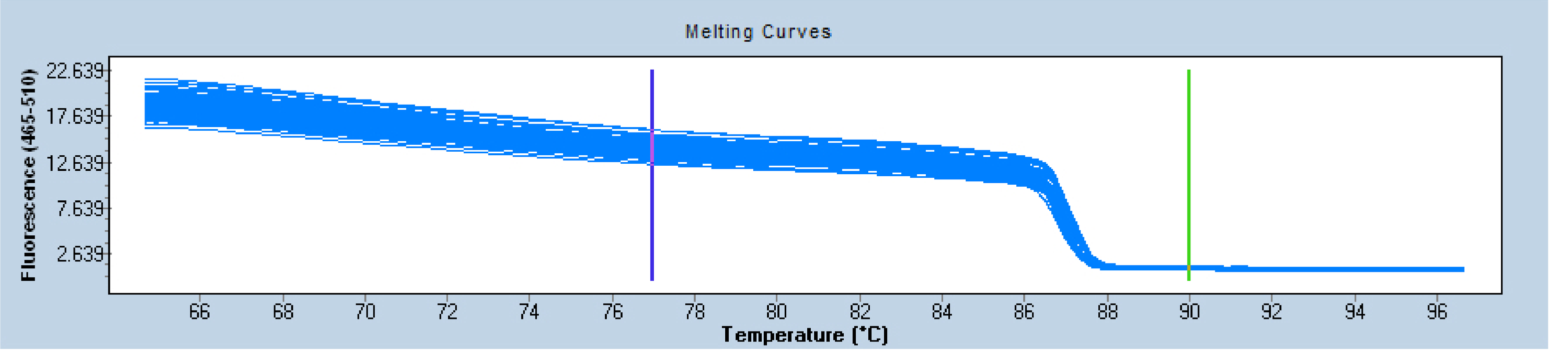

GMNT

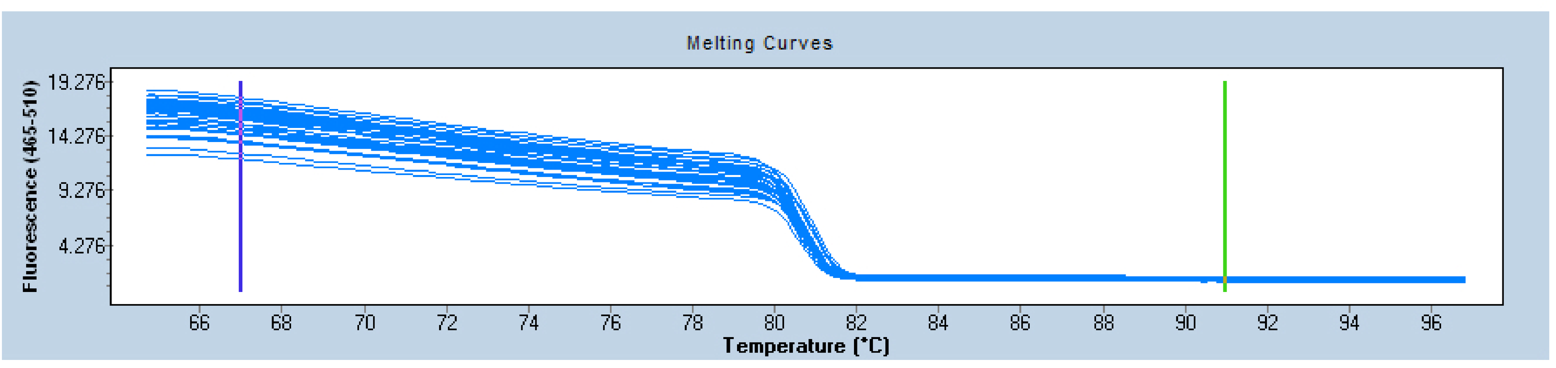

SDC2

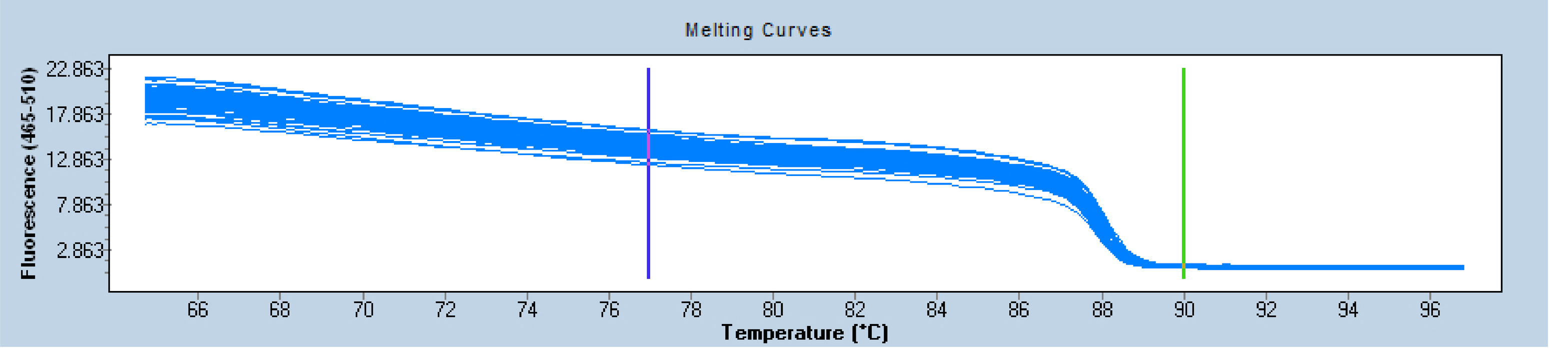

WARS

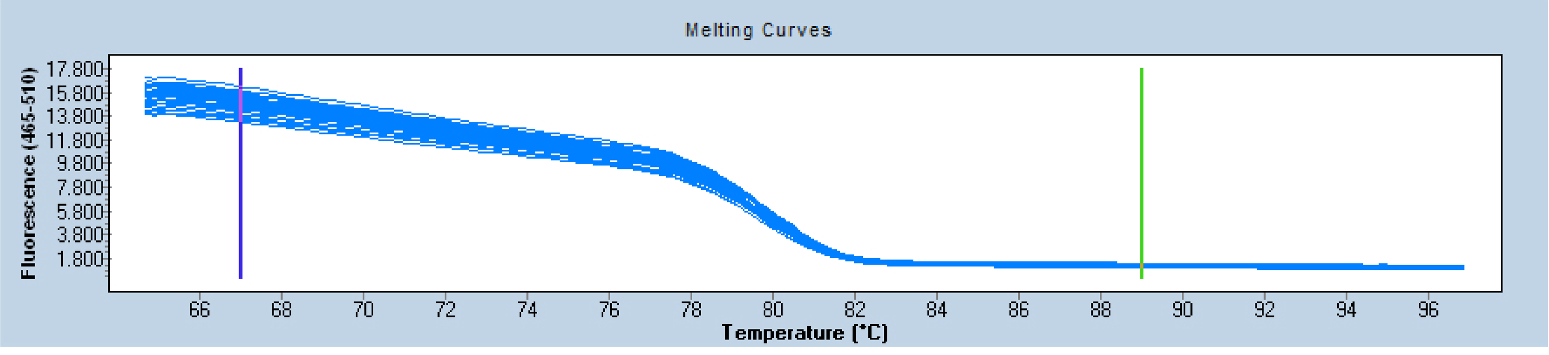

Melting peak chart of lnc/mRNAs selected for RT-qPCR

LNC_002426

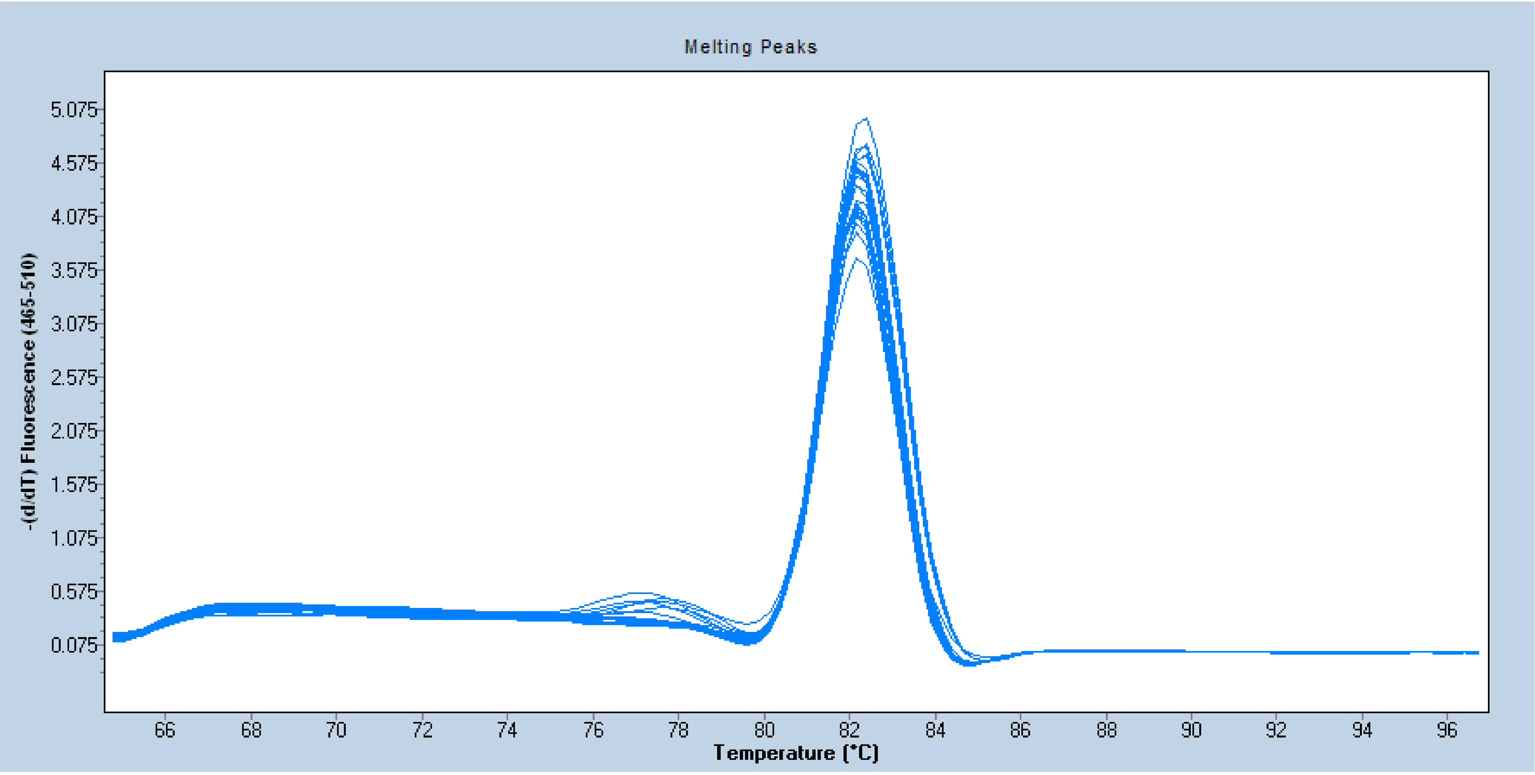

LNC_002540

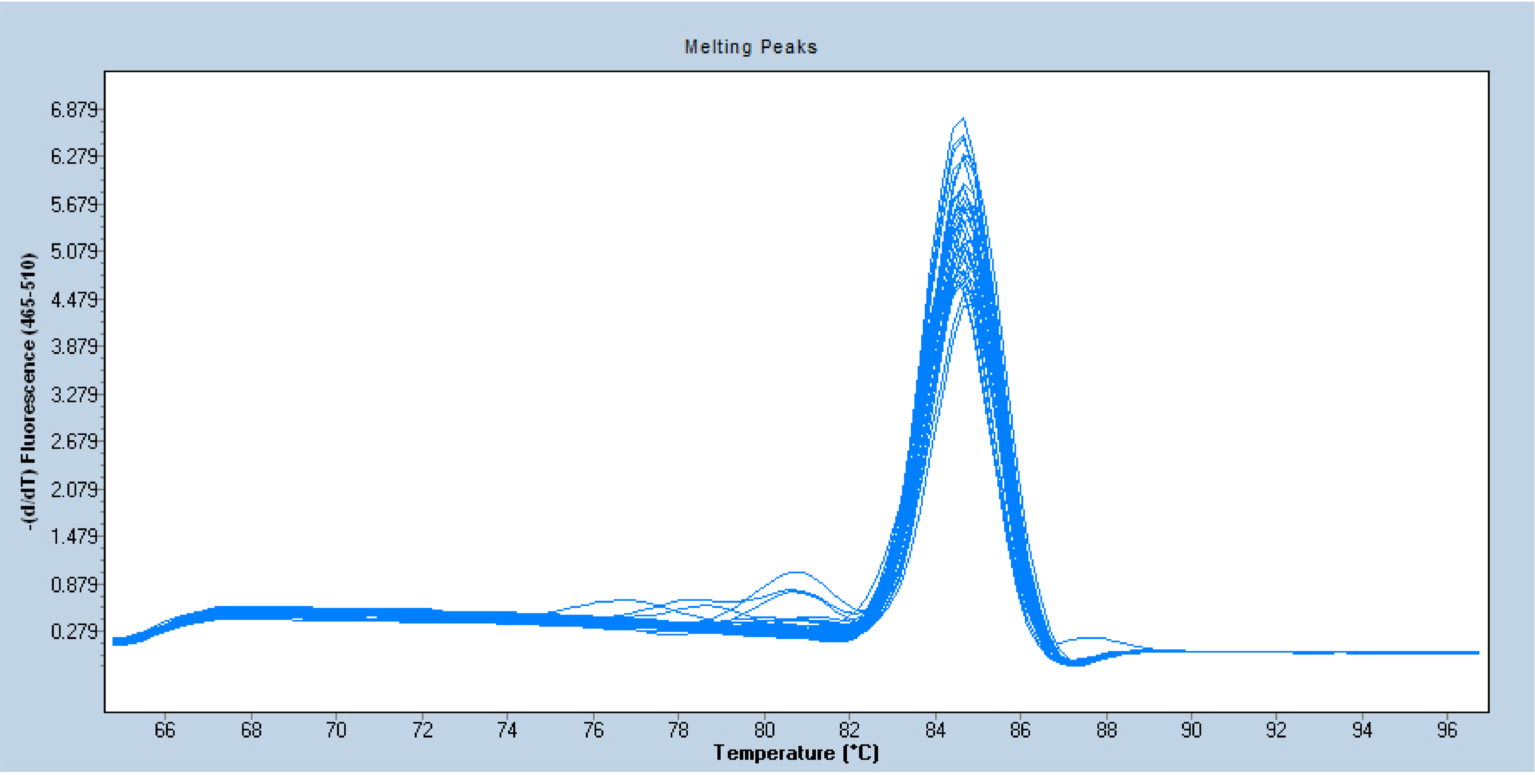

LNC_011254

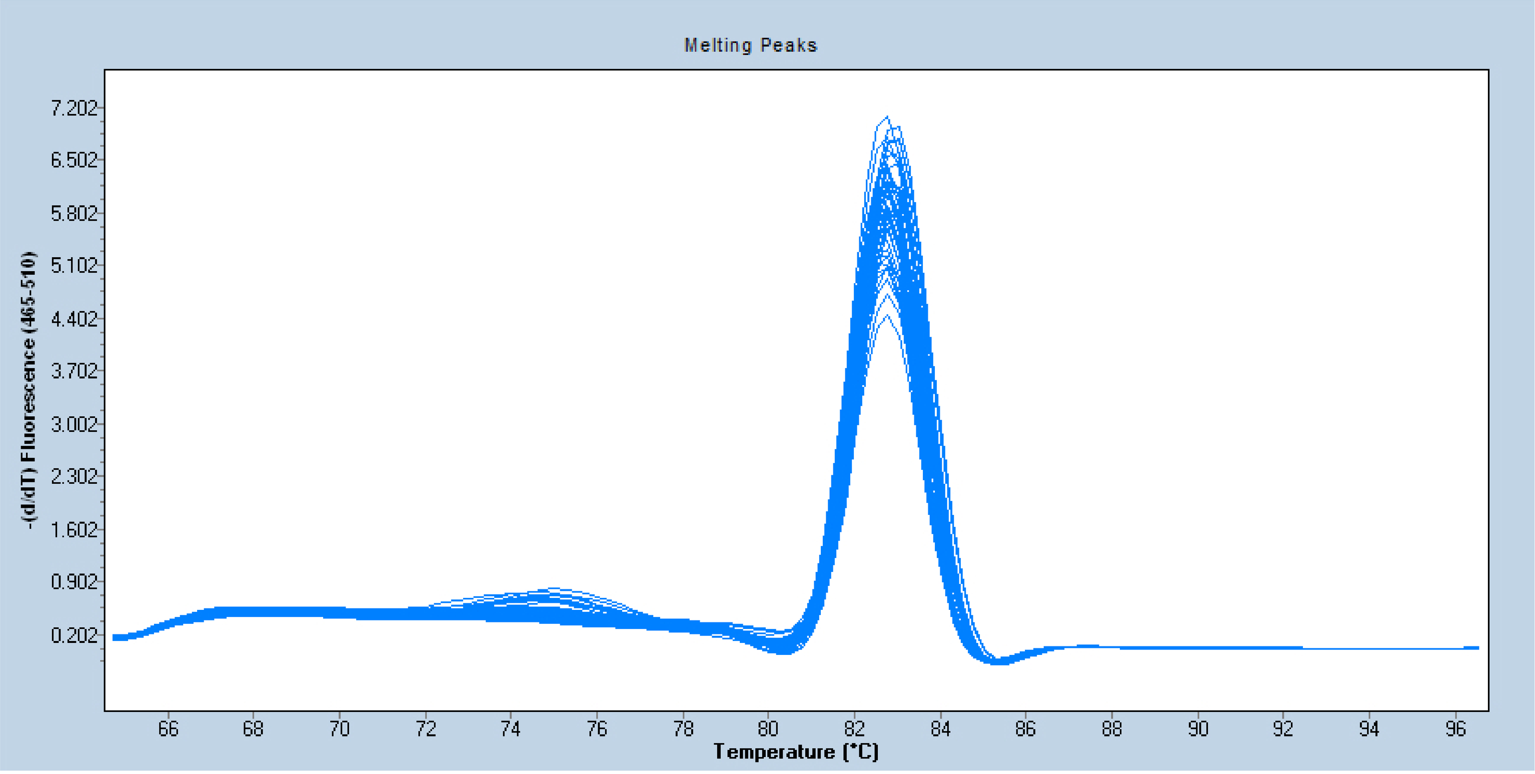

LNC_014194

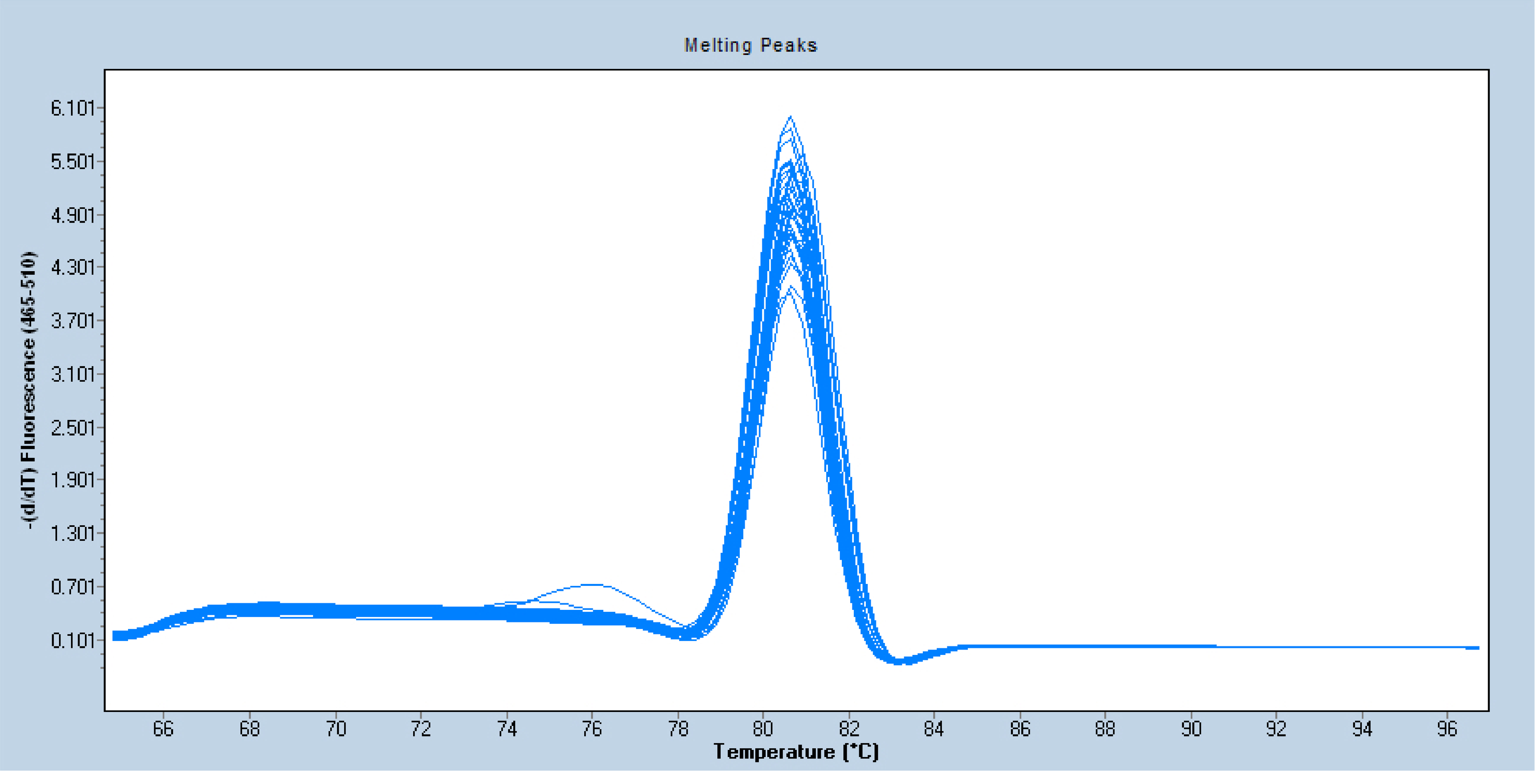

LNC_015020

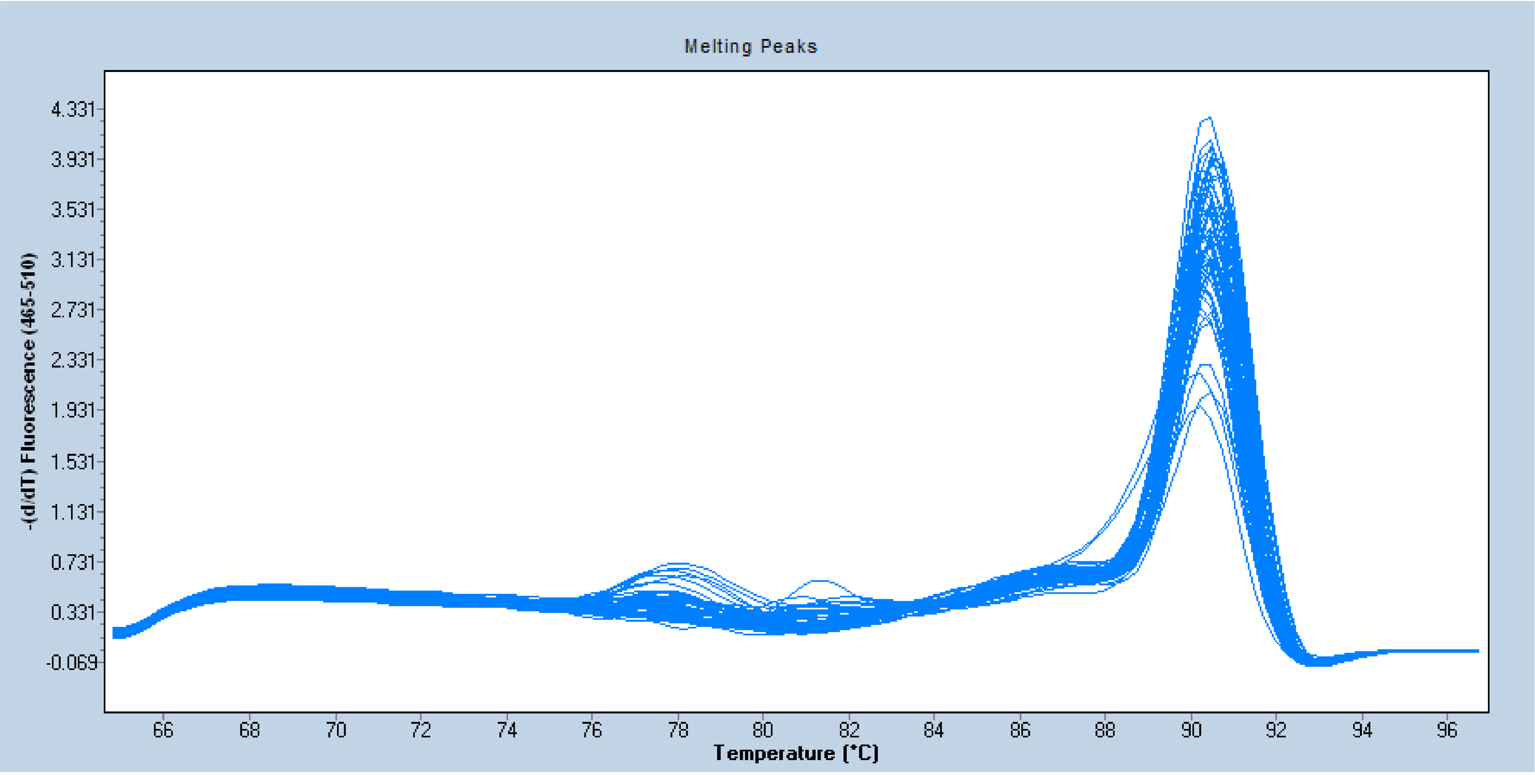

LNC_018655

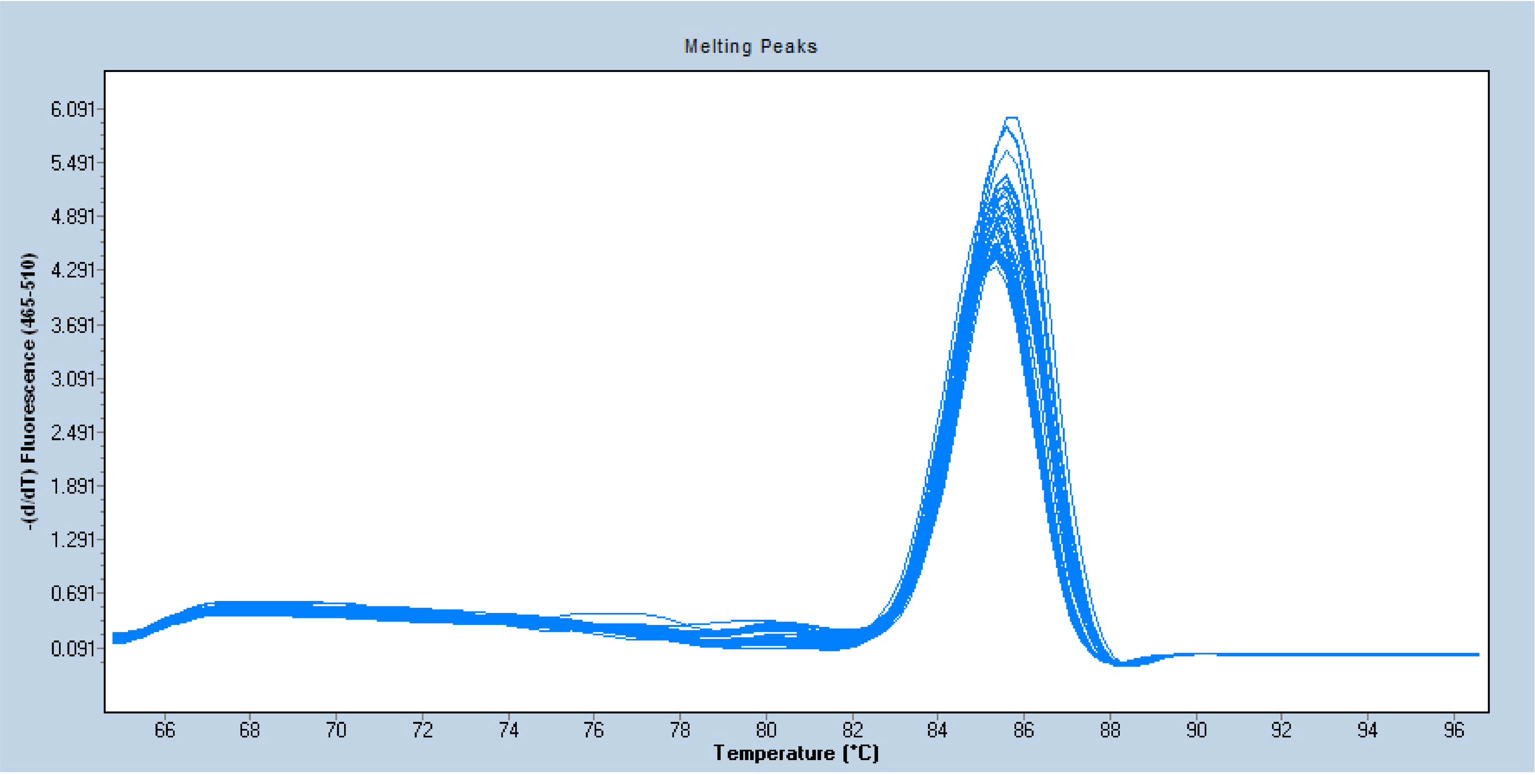

CDCA7

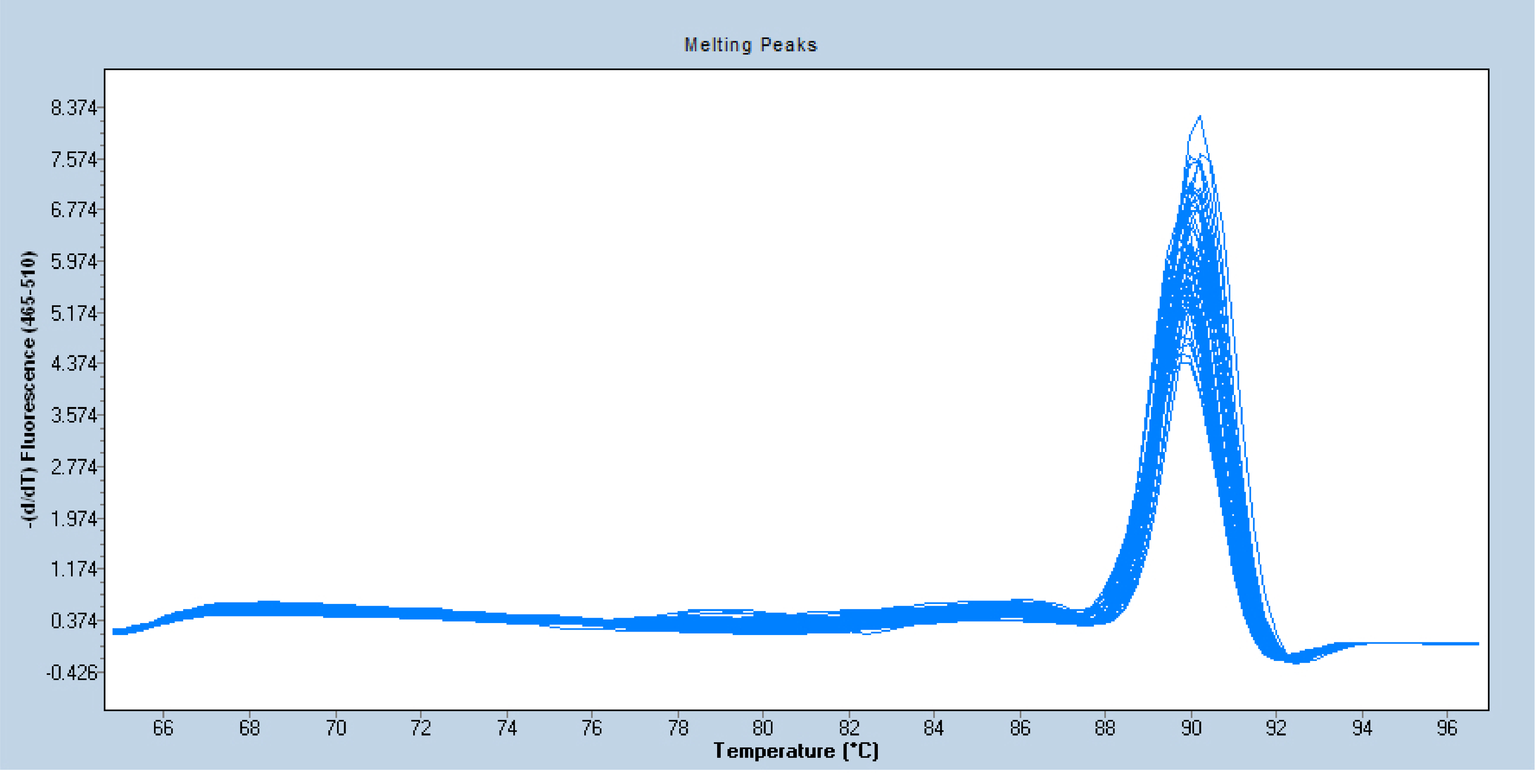

COL3A1

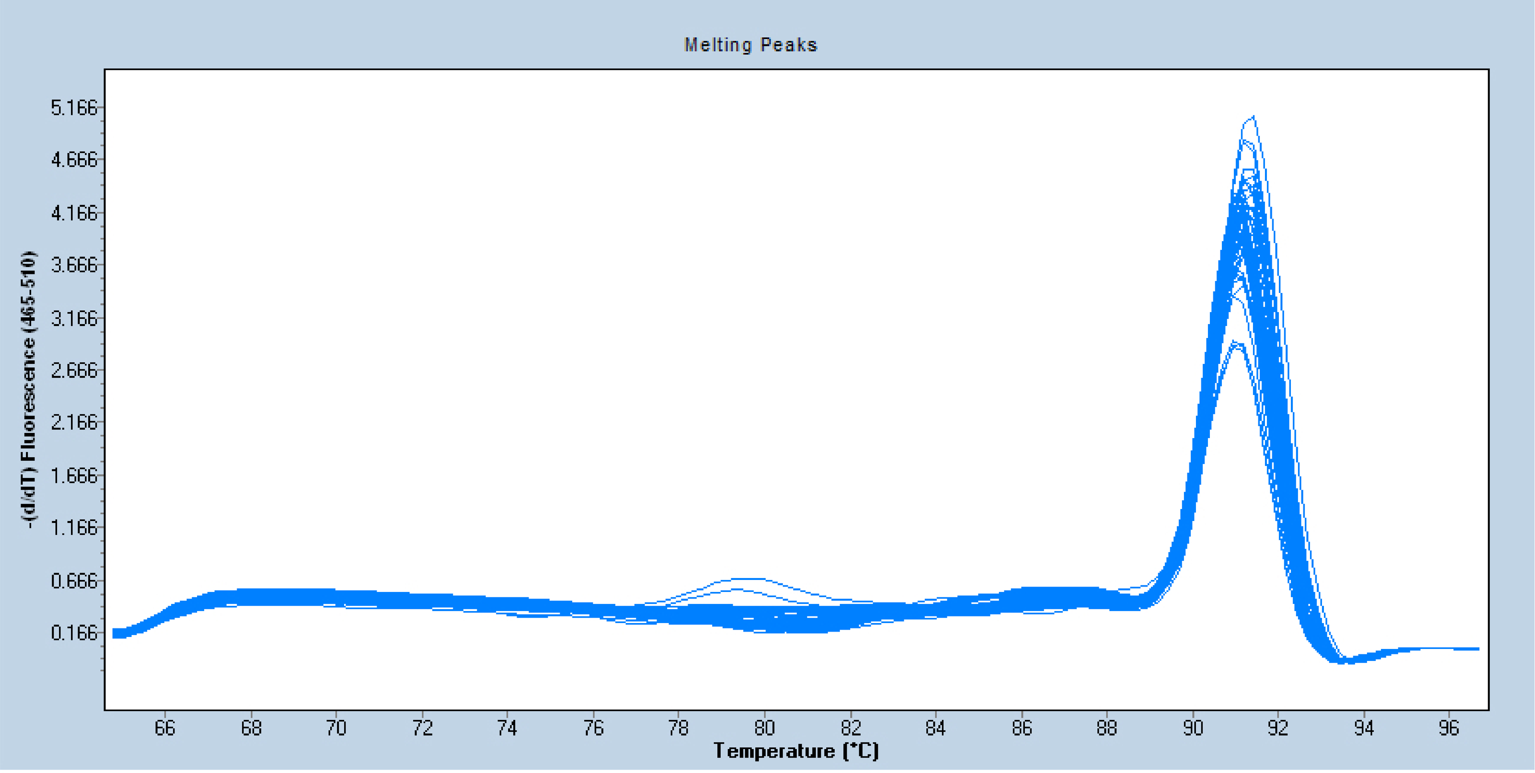

ECM2

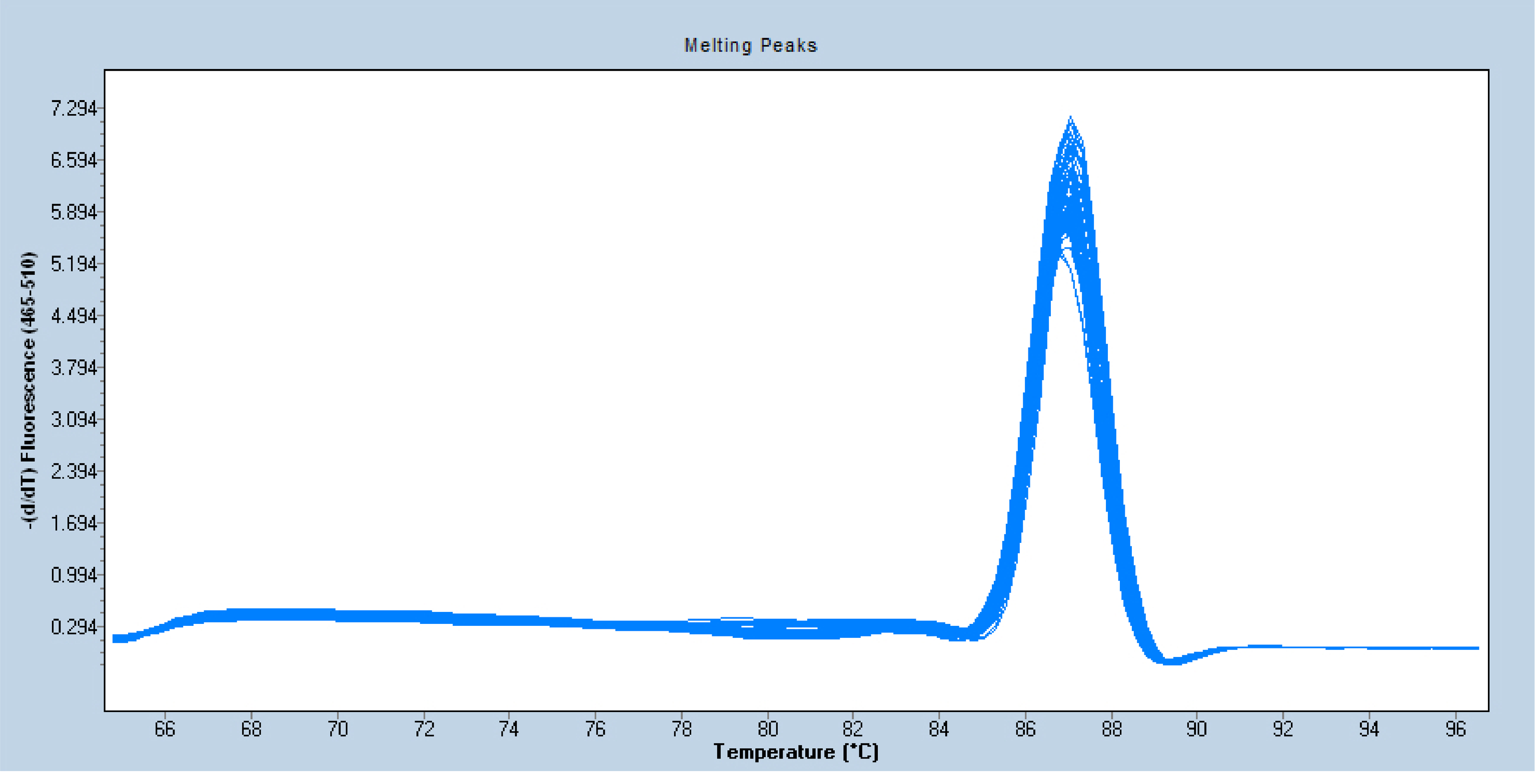

GMNT

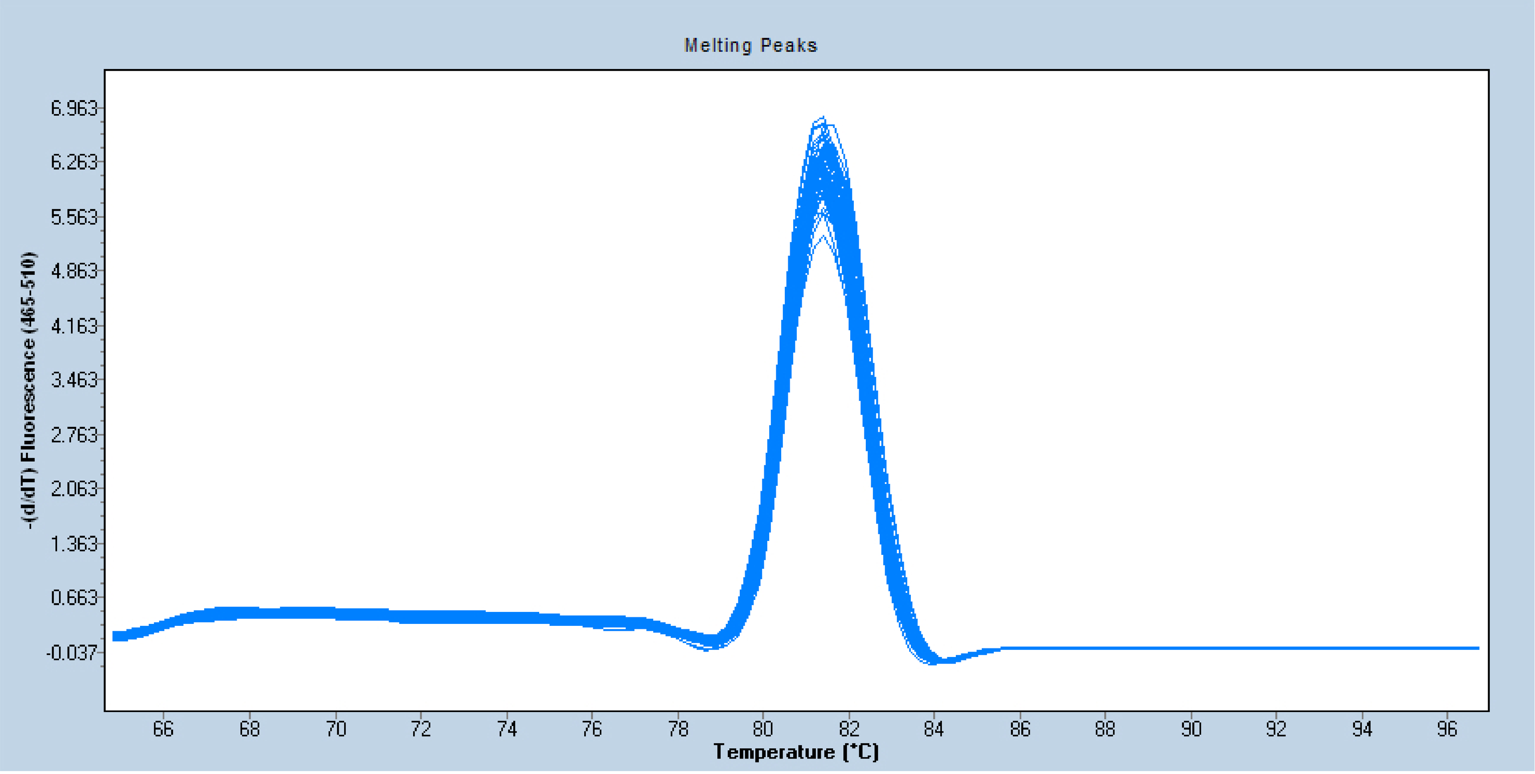

SDC2

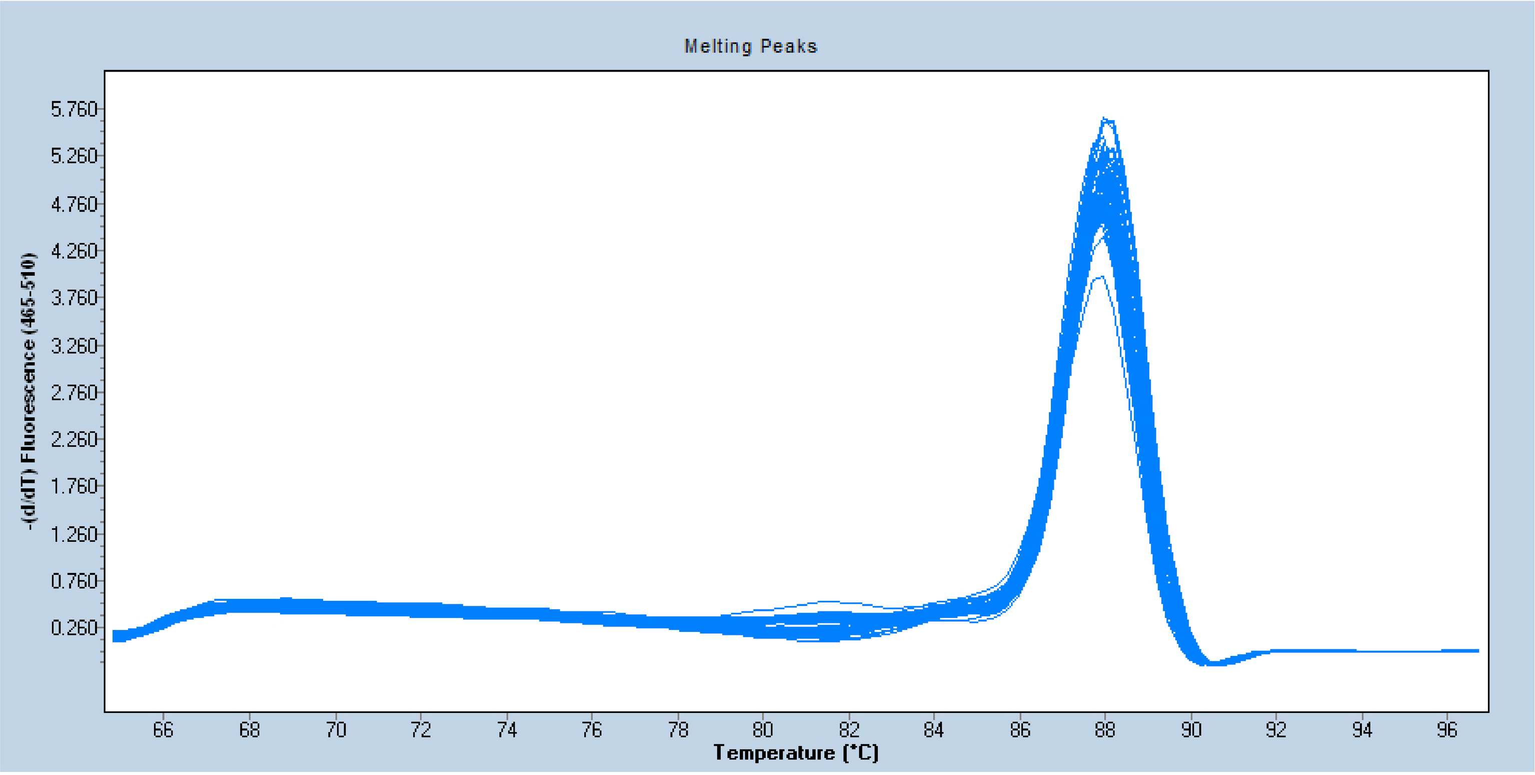

WARS

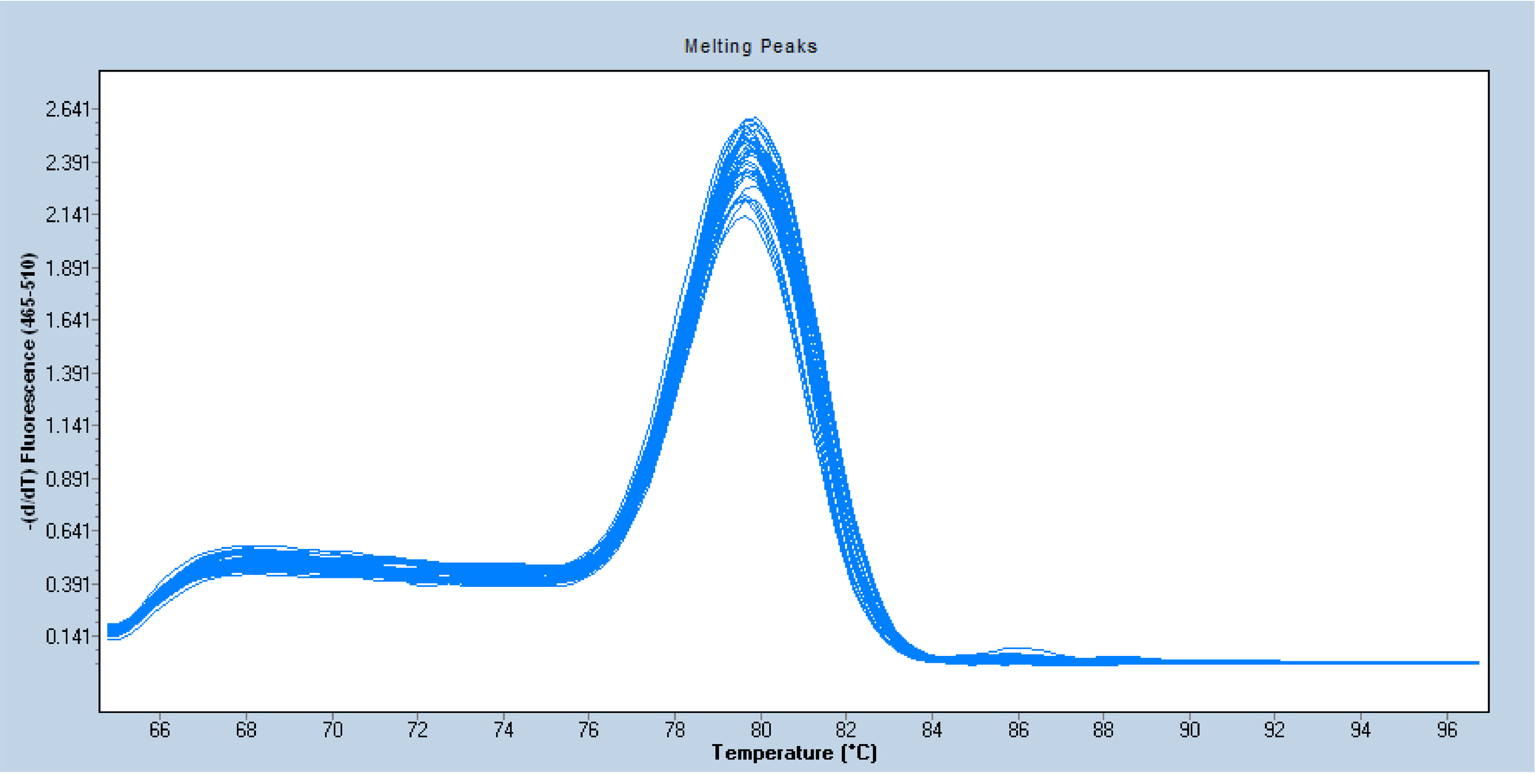

Supplement: FIGURE S2 — Melting curve chart and melting peak chart of RT-qPCR. [file Data_Sheet_2.PDF]
